# Reappraisal on pharmacological and mechanical treatments of heart failure

**DOI:** 10.1186/s12933-020-01024-5

**Published:** 2020-05-06

**Authors:** Bo Liang, Yu-Xiu Zhao, Xiao-Xiao Zhang, Hui-Ling Liao, Ning Gu

**Affiliations:** 1 Nanjing University of Chinese Medicine, Nanjing, China; 2 Hospital (T.C.M.) Affiliated to Southwest Medical University, Luzhou, China; 3 College of Integrated Traditional Chinese and Western Medicine, Southwest Medical University, Luzhou, China; 4 Nanjing Hospital of Chinese Medicine Affiliated to Nanjing University of Chinese Medicine, Nanjing, China

**Keywords:** Heart failure, Pharmacotherapy, Devices, Treatment, Management, Prognosis, Comorbidities

## Abstract

Heart failure (HF) is a highly frequent disorder with considerable morbidity, hospitalization, and mortality; thus, it invariably places pressure on clinical and public health systems in the modern world. There have been notable advances in the definition, diagnosis, and treatment of HF, and newly developed agents and devices have been widely adopted in clinical practice. Here, this review first summarizes the current emerging therapeutic agents, including pharmacotherapy, device-based therapy, and the treatment of some common comorbidities, to improve the prognosis of HF patients. Then, we discuss and point out the commonalities and areas for improvement in current clinical studies of HF. Finally, we highlight the gaps in HF research. We are looking forward to a bright future with reduced morbidity and mortality from HF.

## Background

**Fig. 1:**
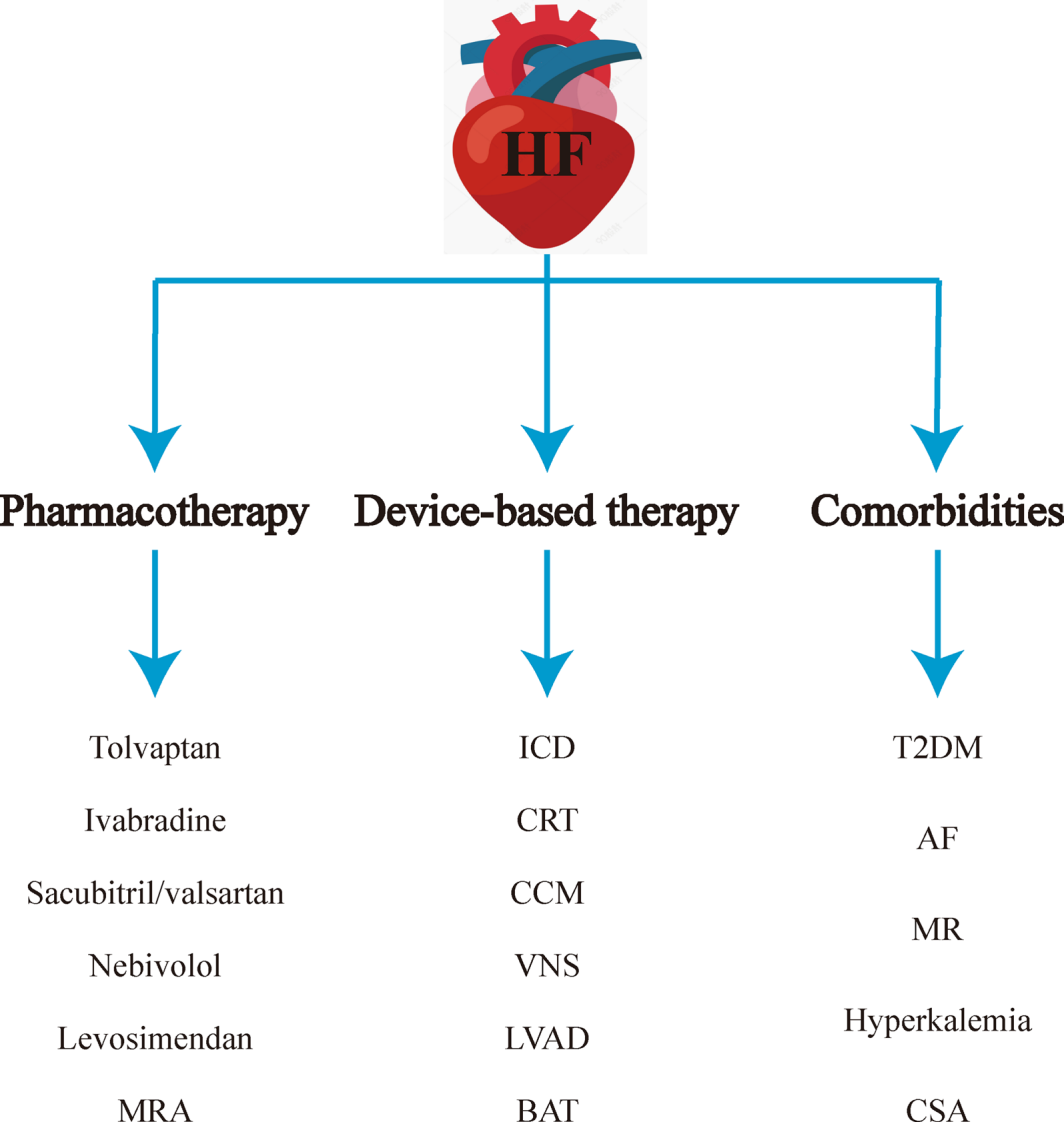
Graphic abstract. *HF* heart failure, *MRA* mineralocorticoid receptor antagonist, *ICD* implantable cardioverter-defibrillator, *CRT* cardiac resynchronization therapy, *CCM* cardiac contractility modulation, *VNS* vagal nerve stimulation, *LVAD* left ventricular assist device, *BAT* baroreflex activation therapy, *T2DM* type 2 diabetes mellitus, *AF* atrial fibrillation, *MR* mitral regurgitation, *CSA* central sleep apnoea

In the past half-century, significant progress has been made in the prevention, diagnosis, and management of cardiovascular diseases. Cardiovascular mortality in developed countries has been reduced by 2/3. The mortality of people with acute coronary syndromes (ACS), valvular, and congenital heart disease, arrhythmia and hypertension has been significantly reduced, with the exception of heart failure (HF). HF is a clinical syndrome that mainly manifests as pulmonary congestion and vena cava congestion, resulting from abnormal cardiac structure and/or function [1]. HF is not an independent disease, but a terminal stage in the development of heart disease. Many diseases can cause HF. In addition to cardiomyopathy and valvular heart disease, cardiogenic diseases also include endocardial or pericardial abnormalities and heart rate (HR) or rhythm disorders. The 2016 guidelines update the classification of HF according to ejection fraction values, including HF with preserved (HFpEF), mid-range (HFmrEF) and reduced ejection fraction (HFrEF) [2]. HF has become a global health burden and affects an estimated 26 million individuals worldwide with a prevalence of approximately 1–2% [3]. The lifetime risk of developing HF is approximately one in five for a 40-year-old in Europe and North America. A total of 74% of HF patients have at least one complication, and such patients are more likely to develop further deterioration of the disease, leading to high hospitalization for HF (HHF) rates and mortality. There is no controversy that HF is currently becoming a preventable and curable disorder based on evidence-based findings [2]. However, the prognosis of advanced HF is worse than that of partial solid tumours and myocardial infarction (MI). There is growing appreciation that the choice of therapy creates exciting new opportunities to improve overall and personalized care, to the individual patient [4]. This review about the reappraisal on pharmacological and mechanical treatments of HF tries to give a compact overview of novel and/or vital trials (Fig. 1) to contribute to a more comprehensive knowledge of this disease.

## Pharmacotherapy

### Tolvaptan

Diuretics are often used in acute HF (AHF) since they can significantly relieve the discomfort of patients, but their side effects are mainly related to electrolyte abnormalities and a deterioration of renal function [5]. Tolvaptan is an oral, selective vasopressin V2 receptor antagonist that induces water excretion and sodium retention. At present, it is often used to treat hypervolemic or isovolumic hyponatraemia with HF, liver cirrhosis and SIADH [6]. Tolvaptan has been shown in many studies to reduce volume load, stabilize haemodynamics, and improve hyponatraemia without affecting renal function. Its application has been recommended by the guidelines for HF [1, 7]. The TACTICS-HF study found that tolvaptan add-on therapy did not improve congestion in AHF [8]. Other studies also indicated that tolvaptan does not affect HR or blood pressure while reducing fluid retention. In addition, in patients with AHF accompanied by renal insufficiency, tolvaptan also has significant benefits [9, 10]. Tolvaptan had no significant effect on potassium or renal function, and it improved the prognosis of AHF patients [11]. EVEREST indicated that the addition of tolvaptan for AHF treatment improved physician-assessed signs and symptoms (including dyspnoea, orthopnoea, fatigue, jugular venous distension, rales, and pedal oedema) during hospitalization without serious adverse short- or long-term effects, but it not reduce long-term cardiovascular morbidity and mortality [12]. In addition to AHF, SECRET and QUEST found that tolvaptan was also effective in chronic HF (CHF) patients. The results from METEOR hold that tolvaptan had no effect on the left ventricular (LV) end-diastolic volume but a significant favourable effect on the composite of mortality and HHF [13]. Tolvaptan corrects hyponatraemia, a predictor of HF. Moreover, tolvaptan produces encouraging changes in filling pressure and significantly increases urine volume in decompensated HF.

### Ivabradine

HR is a modifiable risk factor in HF, and HR acceleration is an independent predictor of the vulnerable phase for HF. In individuals with markedly depressed LV function, the acute administration of ivabradine, the first specific sinus node If channel inhibitor, is well tolerated, effectively reduces HR, markedly increases stroke volume and preserves cardiac output. The results from the SHIFT trial support ivabradine in combination with standard treatment for HF to further improve the symptoms and long-term prognosis [14], and age does not limit the appropriate use of ivabradine [15]. Ivabradine was approved for patients with HF after this trial [7, 16]. The results from INTENSIFY and ETHIC-AHF showed that patients with HF who received early treatment with ivabradine and had better HR control showed significantly improved symptoms of HF and cardiac function. In addition, the early combined use of ivabradine was associated with increased doses of beta-blockers [17], improving exercise tolerance and the quality of life (QoL) [18].

### Sacubitril/valsartan

Sacubitril/valsartan is a dual inhibitor of the angiotensin II receptor and neprilysin. PARADIGM-HF demonstrated that sacubitril/valsartan was preferable to enalapril in lowering the risks of mortality and HHF [19, 20] and led to better health-related QoL in surviving patients [21]. In addition, treatment with sacubitril/valsartan may not only reduce the requirement for loop diuretics [22] but also effectively prevent clinical progression [23] relative to enalapril in HFrEF. These findings of sacubitril/valsartan with stunning interest laid the foundation forrecommendations in guidelines [2, 7, 16]. PIONEER-HF further confirmed the necessity of early use of sacubitril/valsartan in HFrEF [24], and the reduction in N-terminal pro-B-type natriuretic peptide (NT-pro BNP) concentration was weakly yet significantly correlated with reverse cardiac remodelling at 12 months [25]; however, sacubitril/valsartan showed no better reduction in central aortic stiffness than enalapril in HFrEF [26]. An analysis of the TRANSITION study indicated that sacubitril/valsartan has promising results in patients who are naïve to angiotensin-converting enzyme inhibitor (ACEI)/angiotensin receptor blocker (ARB) treatment [27] as well as in patients with de novo HFrEF [28]. Patients in both PIONEER-HF and TRANSITION studies did not use ACEIs/ARBs prior to the initiation of sacubitril/valsartan, suggesting that sacubitril/valsartan and enalapril have similar efficacy and safety in such patients. After seeing significant results in HFrEF, sacubitril/valsartan began to be used in HFpEF studies. In HFpEF patients, the impact of sacubitril/valsartan on NT-pro BNP, left atrial volume, NYHA functional classification and eGFR was independent of SBP reduction [29, 30], but there is no consistent conclusion about clinical events [31]. Studies have revealed that sacubitril/valsartan acts directly on cardiac fibroblasts to prevent maladaptive cardiac fibrosis and dysfunction [32]. Large-sample, multicentre prospective clinical studies are needed to confirm these findings.

### Nebivolol

Large randomized trials have shown that beta-blockers reduce mortality and HHF in HF patients. SENIORS showed that nebivolol (beta1-blocker, NO-releasing activity) is an effective and well-tolerated treatment for HF in elderly patients regardless of ejection fraction [33–35], a subgroup analysis showed nebivolol was less effective in those patients with diabetes [36], and the benefits of nebivolol appeared to be related to the maintenance dose achieved [37]. Nebivolol users had a lower HHF than carvedilol users among concurrent HF and chronic obstructive pulmonary disease patients [38]. Nebivolol might improve cardiac function in HFrEF patients [39], but it did not improve exercise capacity in patients with HFpEF [40]. A pharmacological characteristics study found that lung diffusion and exercise performance (the former likely due to lower interference with β2-mediated alveolar fluid clearance) were higher in patients who received nebivolol [41]. Thankfully, studies involving nebivolol included HF patients, regardless of ejection fraction, but we still need hard endpoint data.

### Levosimendan

In LIDO, levosimendan, a positive inotropic drug with vasodilator effects, improved haemodynamic performance and lowered mortality for up to 180 days compared with dobutamine in severe, low-output HF patients [42]. In the later SURVIVE, levosimendan did not significantly reduce all-cause mortality at 180 days or affect any secondary clinical outcomes, despite an initial reduction in plasma B-type natriuretic peptide (BNP) [43]. REVIVE demonstrated that intravenous levosimendan provided rapid and durable symptomatic relief but was associated with an increased risk of adverse cardiovascular events [44]. LevoRep, a prospective, randomized, double-blind, placebo-controlled, multicentre, parallel-group trial, indicated that intermittent ambulatory treatment with levosimendan in patients with advanced HF did not significantly improve functional capacity or the QoL compared with placebo [45]. Another study showed that levosimendan significantly and safely improved LVEF and the reduced NT-pro BNP of refractory HF in elderly patients [46]. LION-HEART, a multicentre, double-blind, randomized, parallel-group, placebo-controlled trial, found that intermittent administration of levosimendan to outpatients with advanced systolic HF reduced plasma concentrations of NT-pro BNP, worsening of health-related QoL and HHF [47]. For patients with HF complicated by acute MI (AMI), levosimendan treatment improved contractility in post-ischaemic myocardium and was well tolerated without any increase in arrhythmias but with more episodes of hypotension [48]. LEAF studied short-term intravenous infusion of levosimendan, which appears to be more effective than placebo for treating patients with HF complicated by AMI [49]. The result of this confusion may be that the composite endpoint events are not uniform, and LAICA [50] and LeoDOR [51] are warranted to confirm those findings.

### Mineralocorticoid receptor antagonists (MRAs)

Eplerenone is safe and improves clinical outcomes among HFrEF patients in EMPHASIS-HF [52, 53], even in subgroups at high risk of developing hyperkalaemia or worsening renal function [52, 54, 55], and may prevent re-admission when initiated soon after HHF or ACS in patients with systolic HF and mild symptoms [53]. Even moderate prolongation of QRS duration, baseline heart rate, and hypokalaemia were associated with a high risk of worse outcomes in HFrEF, and eplerenone was similarly effective, irrespective of QRS duration/morphology [56], baseline heart rate [57], and hypokalaemia [58]. Moreover, eplerenone improved outcomes in HFrEF patients with and without abdominal obesity [59]. Eplerenone was well-tolerated in Japanese patients with HFrEF and showed results consistent with those reported in the EMPHASIS-HF [60]. In EPHESUS, eplerenone reduced hospitalizations and mortality in HFrEF/HEpEF [61], regardless of percutaneous coronary intervention [62]. However, no relevant studies support the hypothesis that eplerenone has a significantly beneficial effect on glucose homeostasis in patients with HFrEF and either glucose intolerance or diabetes [63].Finerenone was well tolerated and induced a 30% or greater decrease in NT-proBNP levels in a similar proportion of patients to eplerenone [64], and finerenone was well tolerated in Japanese patients in ARTS-HF Japan [65].

In TOPCAT, spironolactone did not significantly reduce the incidence of the primary composite outcome of death from cardiovascular causes, aborted cardiac arrest, or HHF in HFpEF patients [66]. However, a post hoc analysis demonstrated possible clinical benefits with spironolactone in patients with HFpEF from the Americas [67]. The potential efficacy of spironolactone was greatest at the lower end of the LVEF spectrum [68], and spironolactone was not associated with alterations in cardiac structure or function [69]. In Aldo-DHF, spironolactone improved LV diastolic function but did not affect maximal exercise capacity, patient symptoms, or the QoL in patients with HFpEF [70]. However, HFpEF patients are resistant to the beneficial effects of spironolactone on LV diastolic dysfunction [71]. In older adults with stable compensated HFpEF, spironolactone was well tolerated and reduced blood pressure, but it did not improve exercise capacity, the QoL, LV mass, or arterial stiffness [72, 73]. For AHF patients, spironolactone was well tolerated, but it did not improve the primary or secondary efficacy end points [74].

MRAs decrease morbidity and mortality in patients with HF [75]. But the current source of evidence is mainly eplerenone and finerenone for HFrEF, and mainly spironolactone for HFpEF, which might help clinicians in their treatment decisions. The improved LV function observed in those trials is contradictory, and further investigation in larger populations is required. For the uncertainty of spironolactone in the treatment of AHF, EARLIER [76] may give us more inspiration. Additional comparative studies are also required to comprehensively characterize the clinical relevance of the pharmacological differences between MRAs.

## Device-based therapy

In addition to novel, optimized pharmacotherapeutic regimens, interventional and surgical methods have also made remarkable progress in recent decades.

### Implantable cardioverter-defibrillators (ICDs)

ICDs are included among the recommendations for non-surgical device treatment of HFrEF in the guidelines [2]. An ICD is recommended to reduce the risk of sudden death and all-cause mortality in patients with symptomatic HF, and an LV ejection fraction (LVEF) ≤ 35% despite ≥ 3 months of optimal medical therapy, ischaemic heart disease (unless they have had an MI in the prior 40 days) or dilated cardiomyopathy, provided they are expected to survive substantially longer than 1 year with good functional status. SCD-HeFT indicated that ICDs reduced the risk of sudden death in HFrEF patients without diabetes, irrespective of aetiology [77]. In addition, in DANISH, a randomized controlled trial, prophylactic ICD implantation in patients with HFrEF not caused by coronary artery disease was not associated with a significantly lower long-term rate of death from any cause than was usual clinical care [78]. Rates of sudden death declined substantially over time among ambulatory patients with HFrEF who were enrolled in clinical trials [79], a finding that is consistent with a cumulative benefit of evidence-based medications on this cause of death, which might reduce the absolute effect of ICDs on mortality. ICDs have changed the landscape of sudden death prevention in HFrEF; moreover, sudden death accounts for ~ 20% of deaths in HFpEF [80]. If ICDs are to be applied to HFpEF, there must be a coordinated effort to identify and select high-risk patients [81]. We need more evidence about indications for the use of ICDs in specific subgroups (HFmrEF/HFpEF) and the optimal selection of ICD candidates.

### Cardiac resynchronization therapy (CRT)

CRT improves cardiac performance in appropriately selected patients, improves symptoms, and well-being and reduces morbidity and mortality [82, 83]. Theerefore, CRT is also among the recommendations for non-surgical device treatment of HFrEF in the guidelines [2]. CRT is recommended for symptomatic patients with HF in sinus rhythm with a QRS duration ≥ 130 ms and left bundle branch block QRS morphology and in those with an LVEF ≤ 35% despite optimal medical therapy in order to improve symptoms and reduce morbidity and mortality in patients with HFrEF regardless of NYHA class who have an indication for ventricular pacing and high degree atrioventricular block in order to reduce morbidity. The CRT studies involved a different range of LVEF, less than 30% in RAFT [84] and MADIT-CRT [85], less than 40% in REVERSE [86], and less than 50% in BLOCK-HF [87]. In other words, relatively few patients with an LVEF of 35–40% have been randomized, and an individual participant data meta-analysis fortunately suggests no diminution of the effect of CRT in this group [83]. Most other trials have compared defibrillators with CRT to ICDs, and a few have compared pacemakers with CRT to backup pacing. To date, the evidence suggests that no difference in mortality was observed between defibrillators with CRT and pacemakers with CRT [78]. Furthermore, it is important to note that not all patients respond favourably to CRT [82], and QRS morphology or duration may act as a predictor of response to CRT. Trials for HF may be warranted, although this intervention may be useful only for some highly selected patient groups.

### Cardiac contractility modulation (CCM)

CCM is safe, improves exercise tolerance and the QoL in HF patients with ejection fractions between 25% and 45% and narrow QRS complex, and leads to fewer HHF [88–90]. CCM is likely to be cost-effective, provided that the treatment benefit can be maintained beyond the duration of the existing clinical trial follow-up [91]. Current studies indicate that CCM can be used as one of the treatment options for HF patients with a normal QRS complex, but the long-term safety and effectiveness of CCM still need further follow-up studies.

### Vagal nerve stimulation (VNS)

Vagal nerve stimulation as delivered in the NECTAR-HF trial for a 6-month period failed to demonstrate a significant effect on primary and secondary endpoint measures of cardiac remodelling and functional capacity in symptomatic HF patients (LVEF ≤ 35%), but quality-of-life measures showed significant improvement [92]. Later, 18-month results showed that although a favourable long-term safety profile was found, improvements in the efficacy endpoints were not seen with VNS, and a new technique for the detection of acute HR responses to VNS suggests that the recruitment of nerve fibres responsible for HR changes was substantially lower in NECTAR-HF than in preclinical models [93]. The ANTHEM-HF trial showed chronic open-loop autonomic regulation therapy via left- or right-sided VNS continued to be feasible and well-tolerated in patients with HFrEF [94], and improvements in cardiac function and HF symptoms were seen after 6 months of VNS and were maintained at 12 months [95]. INOVATE-HF, a multinational, randomized trial involving 85 centres including 707 patients with chronic symptomatic HF (LVEF ≤ 40%), indicated that VNS did not reduce the rate of death or HF events [96]. Given the inconsistencies in the results so far, more methodologically sound trials are warranted.

### LV assist devices (LVADs)

The use of an LVAD as a bridge-to-transplantation strategy can potentially improve patient survival while waiting for transplantation and allow better allocation of donor hearts [97], but now patients with chronic, refractory HF despite medical therapy can be treated with a permanent implantable LVAD [98]. Despite experiencing more frequent adverse events (such as bleeding and worsening HF), LVAD patients improved more in survival with improved functional status, health-related QoL and depression [99]. Preoperative patient optimization using extracorporeal life support improves outcomes of interagency registry for mechanical-assisted circulatory support level I patients receiving a permanent LVAD [100]; however, fewer data are available for longer-term outcomes.

### Baroreflex activation therapy (BAT)

Increased sympathetic and decreased parasympathetic activity contribute to HF symptoms and disease progression. BAT results in a centrally mediated reduction of sympathetic outflow and increased parasympathetic activity. A study showed that BAT was safe and improved functional status, the QoL, exercise capacity, N-terminal pro-brain natriuretic peptide, and possibly the burden of HHF in HFrEF patients with guideline-directed medical therapy [101]. These effects were most pronounced in patients not treated with CRT [102] and were not major differences between patients with and without coronary artery disease [103]. A later study provided evidence that BAT in HFrEF not only improves haemodynamic and clinical profiles but also exerts profound sympathoinhibitory effects, allowing an almost complete restoration of physiological levels of sympathetic neural function [104].

## Comorbidities and concomitant medical therapy

### Type 2 diabetes mellitus (T2DM)

T2DM is common in patients with HF and is associated with considerable morbidity and mortality [105, 106]. There are many glucose-lowering agents used in patients with HF, showing mixed results. Enhanced recognition of those patients would help guide therapeutic decisions.

#### Sodium-glucose cotransporter-2 inhibitors (SGLT-2is)

The initiation of SGLT-2is, underscoring the potential benefit and risks [107], was related to a lower risk of cardiovascular events, including HHF and death in the cardiovascular disease population [108, 109]. In a cohort, SGLT-2i use compared with dipeptidyl peptidase-4 inhibitor (DPP4i) use was associated with a reduced risk of HF [110]. The promising results of SGLT-2is may be related to the upregulation of the renin-angiotensin-aldosterone system [111]. EMPA-REG OUTCOME [112] is recognized by international guidelines as a landmark study [113]. In the empagliflozin group, there were significantly lower rates of HHF [114], which were achieved by improving diastolic stiffness and hence diastolic function [115] as well as attenuating cardiac fibrosis and improving ventricular haemodynamics [116]. In a real-world population similar to those included in the DECLARE-TIMI 58 study, dapagliflozin was safe and resulted in lower event rates of HHF and cardiovascular mortality than other glucose-lowering drugs [117]. DAPA-HF determined that dapagliflozin lowered the risk of worsening HF or death from cardiovascular causes, added to conventional therapy, in a broad spectrum of HFrEF [118, 119]. Canagliflozin reduced HHF with no statistical evidence of heterogeneity of the treatment effect across the primary and secondary prevention groups [120]. In addition, a number of randomized trials (such as DELIVERED, PRESERVED-HF, and EMPEROR-PRESERVED) are underway to explore the efficacy of SGLT-2is in HFpEF patients. However, because the patients in these studies did not demonstrate any HF-related manifestations or the degree of HF was very low at baseline, any recommendation of SGLT2is for the treatment of HF should be cautious. The current COAPT study included HFrEF and HFmrEF patients, and it is expected to answer this question.

Significant advances have recently occurred in the treatment of T2DM, with evidence of SGLT-2is showing either neutral or beneficial cardiovascular effects. A subanalysis of trials addressing HF treatment in the general population has shown that all HF therapies are similarly effective regardless of T2DM [121]. EMPA-REG OUTCOME demonstrated that patients with T2DM and cardiovascular disease who received empagliflozin, compared with placebo, had a lower rate of the primary composite cardiovascular outcome and of death from any cause when the study drug was added to standard care [112, 122, 123]. The first interim analysis from EMPRISE showed that compared with sitagliptin, the initiation of empagliflozin was associated with a decreased risk of HHF among patients with T2DM as treated in routine care, with and without a history of cardiovascular disease [124]. In patients with T2DM who had or were at risk for atherosclerotic cardiovascular disease, treatment with dapagliflozin robustly resulted in a lower rate of cardiovascular death or HHF [125, 126], and subgroup analysis showed that dapagliflozin reduced HHF in patients with and without HFrEF [127]. Another prospective multicentre trial showed the beneficial effect of dapagliflozin on LV diastolic functional parameters for T2DM patients with HF [128]. CREDENCE and CANVAS supported that canagliflozin significantly reduced major cardiovascular events in patients with T2DM and cardiovascular disease [129–131]. Similarly, in patients with T2DM and a history of HF, canagliflozin reduced the risk of cardiovascular death or HHF [132]. Nevertheless, the evidence of the therapeutic effect of SGLT-2is on patients with HF and T2DM is still insufficient, and long-term clinical testing is needed.

#### Glucagon-like peptide-1 (GLP-1)

Abnormal myocardial metabolism has been documented in the HF population, with reduced fatty acid oxidation and myocardial insulin resistance [133, 134]. GLP-1 increases myocardial insulin sensitivity and has a cardioprotective nature [135–137]. Previous small-sample, retrospective studies showed that GLP-1 was associated with favourable effects that GLP-1 significantly improved LV function, functional status, and the QoL [138]; and that GLP-1 reduced the risk of HHF, all-cause hospitalization, and death in T2DM [139]. Another study involving 32 T2DM patients with a history of CHF provided evidence that treatment with liraglutide is associated with improvements in cardiac function and functional capacity [140]. However, in LEADER, the rate of the primary composite outcome in the time-to-event analysis (the first occurrence of death from cardiovascular causes, nonfatal MI, or nonfatal stroke) among patients with T2DM and high cardiovascular risk was lower with liraglutide than with placebo, whereas the number of HHF was reduced non-significantly in the active arm [141]. Moreover, the unfavourable results observed in LEADER are not stand-alone findings. LIVE, a randomized, double-blinded, placebo-controlled multicentre trial, determined that although the GLP-1 analogue liraglutide did not affect LV systolic function compared with placebo in stable CHF patients with and without T2DM, treatment with liraglutide was associated with an increase in HR and more serious cardiac adverse events [142]. The FIGHT trial, a multicentre, double-blind, placebo-controlled randomized clinical trial of HFrEF, revealed that the use of liraglutide did not lead to greater post-hospitalization clinical stability [143]. In SUSTAIN 6, involving 3297 patients with type 2 diabetes who were at high cardiovascular risk, the rate of cardiovascular death, nonfatal MI, or nonfatal stroke was significantly lower among patients receiving semaglutide than among those receiving placebo, an outcome that confirmed the noninferiority of semaglutide [144, 145].

In summary, the results of the body of existing evidence do not support the use of liraglutide or semaglutide in HF with T2DM, and LIVE points out the potential harmful effect of liraglutide in this population. Importantly, the safety of these powerful GLP-1 analogues in patients with T2DM and documented HF remains uncertain. Therefore, further studies are needed to assess the risks and benefits of liraglutide and semaglutide in this particular subgroup of diabetic patients.

#### DPP4is

TECOS, a randomized, double-blind study assigned 14,671 patients with T2DM and cardiovascular disease, revealed that sitagliptin, a DPP4i, use did not affect the risk of major adverse cardiovascular events, HHF, or other adverse events [146, 147]. Another DPP4i, saxagliptin, increased HHF, independent of ischaemic events. SAVOR-TIMI 53 assigned 16,492 patients with T2DM who had a history of, or were at risk for, cardiovascular events to receive saxagliptin or placebo and followed them for a median of 2.1 years [148]. VIVIDD indicated that compared with placebo, vildagliptin had no major effect on LVEF, but it did lead to an increase in LV volumes in T2DM and HFrEF patients [149]. A secondary analysis of HF and related outcomes with linagliptin versus placebo in CARMELINA, a cardiovascular outcomes trial that enrolled participants with T2DM and atherosclerotic cardiovascular disease and/or kidney disease, demonstrated that linagliptin did not affect the risk of HHF or other selected HF-related outcomes, including among participants with and without a history of HF, across the spectrum of kidney disease, and independent of previous LVEF [150]. A multicentre, double-blind, randomized, placebo-controlled, parallel group, phase III study that assigned 4202 patients with T2DM and established cardiovascular disease indicated that omarigliptin did not increase the risk of cardiovascular death, nonfatal MI, nonfatal stroke or HHF and was generally well tolerated [151].

As treatment with DPP4is exerts no clinically meaningful effects on BNP and NT-proBNP [152], serial monitoring of NT-proBNP in patients with T2DM and ischaemic heart disease may be useful for the identification of patients at highest risk for HF [153]. More evidence is needed regarding the safety of DPP4i in patients with HF and T2DM.

### Atrial fibrillation (AF)

AF and HF are co-evolving epidemics [154]. HF promotes AF, and AF exacerbates HF, which is a vicious circle, and effective treatment is necessary. HF-ACTION illustrated that AF in patients with CHF was associated with older age, reduced exercise capacity at baseline, and a higher overall rate of clinical events [155]. Among HF patients with a history of AF, those with paroxysmal AF were at greater risk of HHF and stroke than those with persistent or permanent AF [156]. AF at enrolment was associated with increased cardiovascular risk in HFpEF patients in the TOPCAT study, and AF was associated with an increased risk of morbidity and mortality [157]. In those special groups, the initiation of digoxin treatment was independently associated with higher mortality [154, 158]. AATAC showed that catheter ablation (CA) of AF was superior to amiodarone in achieving freedom from AF at long-term follow-up and reducing unplanned hospitalization and mortality in patients with HF and persistent AF [159]. CASTLE-AF also discovered that CA was associated with a significantly lower rate of a composite end point of death from any cause or HHF than medical therapy [160]. One study suggested that it may be safe to discontinue oral anticoagulation in post-ablation patients under diligent monitoring, in the absence of AF recurrence, a history of ischaemic stroke/transient ischaemic attack/systemic embolism, and diabetes mellitus [161, 162], but evidence is lacking in patients with HF and AF. The choice of optimal treatment strategies for patients with both AF and HF is increasingly difficult, given that results from trials of pharmacological rhythm control are arguably obsolete in the age of CA. Restoring sinus rhythm by CA seems successful. In addition to GENETIC-AF [163], long-term studies to examine the effect on HHF, stroke, and death are warrannted.

### Mitral regurgitation (MR)

In patients who have HFrEF, severe secondary MR is associated with a poor prognosis [164]. The MITRA-FR trial showed that among patients with severe secondary MR, the rate of death or unplanned HHF at 1 year did not differ significantly between patients who underwent percutaneous mitral-valve repair in addition to receiving medical therapy and those who received medical therapy alone [165]. The same goes for the 24-month outcome from the MITRA-FR trial [166]. Instead, the COAPT trial concluded that among patients with HF and moderate-to-severe or severe secondary MR who remained symptomatic despite the use of maximal doses of guideline-directed medical therapy, transcatheter mitral-valve repair resulted in a lower rate of HHF and lower all-cause mortality within 24 months of follow-up than medical therapy alone, and the rate of freedom from device-related complications exceeded a prespecified safety threshold [167]. We are looking forward to more similar research, such as RESHAPE-HF-2, MATTERHORN, EVOLVE-HF and CLAMP [168], to explore whether percutaneous mitral-valve repair improves clinical outcomes in this patient population.

### Hyperkalaemia

Chronic kidney disease (CKD) in HF increases the risk of hyperkalaemia [169]. The PEARL-HF trial holds that patiromer (a non absorbed, orally administered, potassium-binding polymer) prevented hyperkalaemia and was relatively well tolerated in patients with HF and a history of hyperkalaemia, resulting in the discontinuation ofrenin-angiotensin-aldosterone system (RAAS) inhibitors and/or beta-adrenergic blocking agents, or in CKD patients with an estimated glomerular filtration rate of < 60 mL/min receiving standard therapy and spironolactone (25–50 mg/day) [170]. In patients with a clinical diagnosis of HF, diabetes, CKD, and hyperkalaemia on RAAS inhibitors, patiromer was well tolerated and effective for hyperkalaemia treatment over 52 weeks [171], as shown in results from AMETHYST-DN. In addition, new evidence is available from Patiromer-204, an open-label study, in which patiromer followed by individualized titration maintained serum potassium within the target range in the majority of patients with HF and CKD, all of whom were uptitrated to spironolactone 50 mg/day. Patiromer was well tolerated, with a low incidence of hyperkalemia, hypokalaemia, and hypomagnesemia [172]. Lokelma was well-tolerated in patients with stable CKD and hyperkalaemia [173] and in those with predialysis hyperkalaemia with end-stage renal disease undergoing adequate hemodialysis [174]. Lokelma enables substantial RAAS inhibitors to change while achieving normokalaemia [175]. However, no new evidence is available for lokelma in the field of HF. We need more evidence (PRIORITIZE HF and DIAMOND) to further confirm this.

### Central sleep apnoea (CSA)

Periodic breathing with CSA is common in HF and is associated with poor QoL and increased risk of morbidity and mortality [176]. Adaptive servo-ventilation had no significant effect on the primary end point in patients who had HFrEF and predominantly CSA, but all-cause and cardiovascular mortality were both increased with this therapy, as seen in results from SERVE-HF [177]. The use of chronic transvenous phrenic nerve stimulation appeared to be safe and feasible in HF patients with CSA [178–180]. This approach may represent a novel therapy for CSA and warrants further prospective, randomized, controlled trials.

## Discussion

HF is an important cardiovascular disease because of its increasing prevalence, significant morbidity, high mortality, and rapidly expanding health-care costs. According to the Global Burden of Diseases, Injuries, and Risk Factors Study 2017, there are more than 60 million HF patients worldwide [181]. As the life expectancy of the population increases and our ability to diagnose and treat cardiovascular diseases such as ACS increases, it is estimated that these numbers will continue to increase [182]. It is estimated that the lifetime risk of HF in adults ranges from 20 to 45% [183]. The risk of rehospitalization and cardiovascular death for HF hospitalized patients is increased [184]. One in 8 deaths results from HF, with a mortality rate of up to 50% within 5 years of diagnosis [184]. In addition to the heavy burden of disease, HF also brings a large economic burden. The total global cost of HF was estimated at 108 billion dollars in 2012 [185], and this number is expected to increase significantly over time. In the United States, the cost of HF is expected to increase by 127% by 2030 [184].

HF has underlying causes, pathophysiological complexities, and concomitant comorbidities, which make both diagnosis and treatment particularly challenging. Coupled with rising risk factors such as hypertension, T2DM, and obesity, HF requires more consideration by medical staff and patients and their families. Here, we have summarized the treatments of and corresponding key trials in HF (Table 1). However, looking at HF as a life-limiting syndrome makes it possible to provide multidisciplinary patient-centred care focused on both extending survival when possible and achieving the best possible QoL.

**Table 1:** Summary of treatments and corresponding key trials

Treatments	Key trials
Pharmacotherapy	
Tolvaptan	TACTICS-HF [8], AQUAMARINE [9, 10], Shirakabe et al. [11], EVEREST [12], SECRET, QUEST, METEOR [13]
Ivabradine	SHIFT [14, 15], INTENSIFY, ETHIC-AHF, Bagriy et al. [17], CARVIVA HF [18]
Sacubitril/valsartan	PARADIGM-HF [19–23], PIONEER-HF [24, 25], EVALUATE-HF [26], TRANSITION [27, 28], PARAMOUNT [29, 30], PARAGON-HF [31]
Nebivolol	SENIORS [33–37], Sessa et al. [38], Brehm et al. [39], ELANDD [40], CARNEBI [41]
Levosimendan	LIDO [42], SURVIVE [43], REVIVE [44], LevoRep [45], Zhang et al. [46], LION-HEART [47], LEAF [48], Jia et al. [49], LAICA [50], LeoDOR [51]
MRA	Eplerenone: EPHESUS [75], EMPHASIS-HF [52–59], J-EMPHASIS-HF [60], EPHESUS [61, 62], EARLIER [76] Finerenone: ARTS-HF [64], ARTS-HF Japan [65] Spironolactone: TOPCAT [66–69, 201], Aldo-DHF [70, 71], Upadhya et al. [72], ATHENA-HF [74]
Device-based therapy	
ICD	SCD-HeFT [77], DANISH [78].
CRT	RAFT [84], MADIT-CRT [85], REVERSE [86], BLOCK-HF [87]
CCM	FIX-HF-5 [88, 89, 91], Borggrefe et al. [90].
VNS	NECTAR-HF [92, 93], ANTHEM-HF [94, 95], INOVATE-HF [96]
LVAD	ROADMAP [99], INTERMACS [100].
BAT	HOPE4HF [101], Zile et al. [102], Halbach et al. [103], Raffaella et al. [104]
Comorbidities	
T2DM	SGLT-2i: Empagliflozin EMPA-REG OUTCOME [112, 114, 122], EMPRISE [124]; Dapagliflozin: DECLARE-TIMI 58 [117, 125–127], Soga et al. [128]; DAPA-HF [118, 119]; Canagliflozin: CANVAS [120, 131, 132], CREDENCE [129, 130]. GLP-1: Liraglutide: LEADER [141], LIVE [142], and FIGHT [143]; Semaglutide: SUSTAIN-6 [144] DPP4i: Sitagliptin: TECOS [146, 147]; Saxagliptin: SAVOR-TIMI 53 [148]; Vildagliptin: VIVIDD [149]; Linagliptin: CARMELINA [150]; Omarigliptin: Gantz et al. [151]
AF	ENGAGE AF-TIMI 48 [154], HF-ACTION [155], PARADIGM-HF/ATMOSPHERE [156], TOPCAT [157], ARISTOTLE [158], AATAC [159], CASTLE-AF [160], GENETIC-AF [163]
MR	MITRA-FR [165, 166], COAPT [167]
Hyperkalemia	Patiromer: PEARL-HF [170], AMETHYST-DN [171], Patiromer-204 [172] Lokelma: Stephen et al. [173], DIALIZE [174], ZS-005 [175]
CSA	SERVE-HF [177], Ponikowski et al. [178], Pilot [179], Zhang et al. [180]

### Challenges and limitations in the current treatment of HF

**Fig. 2:**
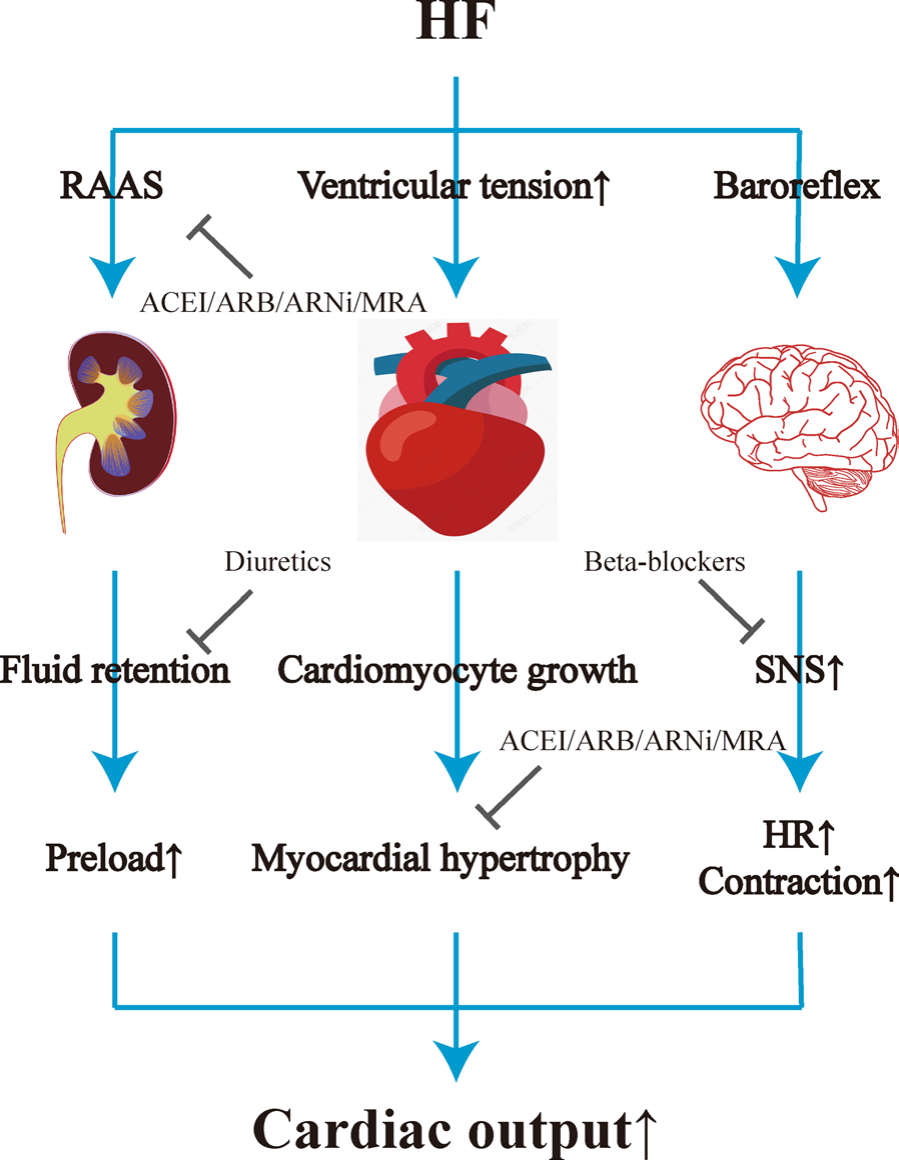
The conventional drug treatment of HF. *HF* heart failure, *RAAS* renin-angiotensin-aldosterone system, *ACEI* angiotensin-converting enzyme inhibitor, *ARB* angiotensin receptor blocker, *ARNi* angiotensin receptor-neprilysin inhibitor, *MRA* mineralocorticoid receptor antagonist, *SNS* sympathetic nervous system, *HR* heart rate

We are making progress, but it has been extraordinarily slow. Over the past three decades, with the passage of time, the clinical treatment of HF has continued to increase. Although the mortality rate of HF has shown a downward trend, it is still at a high level. Numerous studies have shown that patients with HF still have a high residual risk despite many approved, guideline-recommended treatments [19, 119]. Therefore, under the conventional drug treatment of intervention in the RAAS and sympathetic nervous system (Fig. 2), the management of HF cannot be met. Diuretics, ACEIs/ARBs, and beta-blockers are the three standard treatments recommended in the guidelines for the treatment of HF. In addition, sacubitril/valsartan is also widely used in clinical practice, but the recommendations for the drug are slightly different among different guidelines [2, 7]. Since 2013, the guidelines have focused on underlying conditions, which may contribute to diastolic dysfunction, such as hypertension and AF. By 2016, the diagnosis of HF had become more sophisticated, but the diagnosis and theatments of HFmrEF/HFpEF are more challenging than those of HFrEF. Mortality rates in HFpEF patients are higher than in age- and comorbidity-matched non-HF controls and lower than in HFrEF patients [81], and sudden death accounts for ~ 20% of deaths in HFpEF [80]. Indeed, the management of HFpEF remains problematic due to the paucity of data on the clinical benefits of current therapies, which focus on symptom relief and the reduction of HHF by controlling fluid retention and managing risk factors and comorbidities [81]. PARADIGM-HF [19] triggered hope for the potential benefit of sacubitril/valsartan in HFpEF. However, these hopes were dashed by PARAGON-HF [31]. PARAGON-HF directed us to design trials to test whether a borderline EF is a signal of potential benefit from therapy targeting HFrEF and HFpEF simultaneously [186]. To date, a high-quality clinical trial specifically designed for HFpEF is still lacking, whether it is pharmacotherapy or device-based therapy. Some results on HFpEF have been obtained only through subgroup analysis. Moreover, no prospective trial has been conducted in patients with HFmrEF to date. All analyses are based on post hoc analyses from HFrEF and/or HFpEF trials, with inclusion criteria that included patients now classified as HFmrEF.

In view of increasing interest in diabetes in HF, glucose-lowering agents merit further study in this context. In studies about SGLT-2is, many of the patients included did not have HF even at baseline, and studies focused on HF may be urgent. Additionally, these sources of evidence are the results of randomized double-blind placebo comparisons, and the long-term prognosis is still controversial. Therefore, long-term clinical follow-up and methodologically sound, positive drug control trials are needed to show more valuable information on glucose-lowering agents.

### Future directions in finding novel treatments for HF

HFmrEF/HFpEF is a highly heterogeneous disease both in mechanism and in clinical practice, which may be the reason why many conventional HF treatments fail to achieve good results. The most appropriate strategies for the treatment of HFmrEF/HFpEF have not been defined [187], and we have to consider shifting our attention to less well-investigated hypotheses. The PARAGON-HF [31] study confirmed that sacubitril/valsartan did not improve outcomes in HFpEF, but benefits were found in some special populations, which suggests that we need to find a target-based HFpEF typing method to implement targeted and accurate treatment. Moreover, we need more dedicated studies composed of HFpEF patients, rather than analyses of patient subgroups derived from preexisting trials, which introduce confounding biases and generalization difficulties.

Exciting research into new biological targets is currently underway. The activation of the cyclic guanosine monophosphate pathway is among those avenues, with several studies examining the roles of udenafil and BAY1021189 in the treatment of HFpEF [188]. In SOCRATES-REDUCED, among patients with worsening chronic HFrEF, compared with placebo, vericiguat, an oral soluble guanylate cyclase stimulatoror, did not have a statistically significant effect on changes in NT-pro BNP levels but it was well-tolerated [189]. In SOCRATES-PRESERVED, vericiguat also did not change NT-pro BNP levels and left atrial volume, but it was well-tolerated and associated with improvements in the QoL in patients with HFpEF [190]. Based on the results of SOCRATES-REDUCED [189] and SOCRATES-PRESERVED [190], VICTORIA [191], a multicentre, randomized, double-blind, placebo-controlled trial of the efficacy and safety of the oral soluble guanylate cyclase stimulator vericiguat was carried out among a broadly generalizable high-risk population of three unique clinical strata of patients with worsening chronic HFrEF despite very good HF therapy [192]. The latest results showed that the incidence of death from cardiovascular causes or HHF was lower among those who received vericiguat than among those who received placebo [193]. In addition, the REDUCE LAP-HF study, an open-label, single-arm, phase I study designed to assess the performance and safety of a transcatheter interatrial shunt device in patients older than 40 years of age with symptoms of HFpEF despite pharmacological therapy, showed that the implantation of an interatrial shunt device is feasible, seems to be safe, reduces left atrial pressure during exercise, and could be a new strategy for the management of HFpEF [194].

In addition to T2DM, MR, hyperkalaemia, and CSA, HF is also fraquently associated with other diseases, including anaemia, poor renal function, iron deficiency, etc., which is also a direction that needs our continuous exploration. Emphasis should be given to the understanding of comorbidities. SCD-HeFT indicated that ICDs reduced the risk of sudden death in HFrEF patients without diabetes but not in patients with T2DM [77]. There are many trials on HF combined with diabetes, and we urgently need to include this specific population at the beginning of the trials, not through subgroup analysis or post hoc analysis, especially for SGLT-2is. SAFE-IRON-HF and SLEEP-HF were well-positioned to evaluate patients with HF complicated by mild iron deficiency anaemia or sleep disordered breathing.

Exercise is seen as a diagnostic and prognostic tool as well as a therapeutic intervention in CHF [195]. Several trials have shown that exercise is beneficial for CHF [196–200], not only improving functional capacity and the QoL but also reducing the risk of rehospitalization, even showing a tendency towards better survival rates. Moreover, both poor and intermediate self-reported physical activity were associated with a higher risk of HHF and mortality [201]. Peak oxygen uptake and the 6-min walk test may be suitable surrogate end points for the treatment effect of exercise on mortality and the QoL in HF. Different exercise programmes (intensity, frequency) also need further research. Future studies should aim to achieve a consensus on the definition of outcomes and the uniformity of exercise programmes and promote the reporting of a core set of HF data.

Moreover, drug interactions may be important because this is an actual problem in HF due to the inclusion of new drugs in treatment. There is a lack of research on device-based therapy for HF combined with other diseases. This large gap also needs to be filled.

Robust evidence from prospective studies is lacking for most therapies and additional studies will provide further insights into these patient populations. Equally important, however, is the standardization of research protocols (more standardized diagnostic approaches, homogenous exclusion criteria, and HFmrEF/HFpEF-dedicated cohort analyses), the application of which may help improve our understanding of the disease and translate into better outcomes. There is an urgent need to develop evidence-based treatment algorithms that not only alleviate the symptoms of patients and reduce the burden of hospitalization but also, more importantly, increase the QoL and prolong life when possible and in accordance with patient preferences. Although the path to the treatment of HFmrEF/HFpEF is still full of obstacles, we are seeing light at the end of the tunnel.

## Conclusions

Although HF frequently exists, there are still numerous unanswered questions about the pathophysiology, symptomatology, diagnosis, and prognosis. We have barked up this tree for a few decades; it is time to move on. More systematic research is urgently needed to answer these unresolved issues and to provide treatments that can improve the QoL and reduce adverse clinical outcomes in the rapidly expanding number of patients with HF, especially HFmrEF/HFpEF.

## Data Availability

Not applicable.

## Abbreviations

ACEI: Angiotensin-converting enzyme inhibitor
AF: Atrial fibrillation
AHF: Acute heart failure
AMI: Acute myocardial infarction
ARB: Angiotensin receptor blocker
BAT: Baroreflex activation therapy
BNP: B-type natriuretic peptide
CA: Catheter ablation
CCM: Cardiac contractility modulation
CHF: Chronic heart failure
CKD: Chronic kidney disease
CRT: Cardiac resynchronization therapy
CSA: Central sleep apnoea
DPP4i: Dipeptidyl peptidase-4 inhibitor
GLP-1: Glucagon-like peptide-1
HF: Heart failure
HFmrEF: Heart failure with mid-range ejection fraction
HFpEF: Heart failure with preserved ejection fraction
HFrEF: Heart failure with reduced ejection fraction
HHF: Hospitalization for heart failure
HR: Heart rate
ICD: Implantable cardioverter-defibrillator
LV: Left ventricular
LVAD: Left ventricular assist device
LVEF: Left ventricular ejection fraction
MI: Myocardial infarction
MR: Mitral regurgitation
MRA: Mineralocorticoid receptor antagonist
NT-pro BNP: N-terminal pro-B-type natriuretic peptide
QoL: Quality of life
RAAS: Renin-angiotensin-aldosterone system
T2DM: Type 2 diabetes mellitus
VNS: Vagal nerve stimulation

## References

[1] Yancy CW, Jessup M, Bozkurt B, Butler J, Casey DE, Drazner MH, Fonarow GC, Geraci SA, Horwich T, Januzzi JL (2013). 2013 ACCF/AHA guideline for the management of heart failure: a report of the American College of Cardiology Foundation/American Heart Association Task Force on practice guidelines. Circulation.

[2] Ponikowski P, Voors AA, Anker SD, Bueno H, Cleland JGF, Coats AJS, Falk V, González-Juanatey JR, Harjola VP, Jankowska EA (2016). 2016 ESC Guidelines for the diagnosis and treatment of acute and chronic heart failure: the Task Force for the diagnosis and treatment of acute and chronic heart failure of the European Society of Cardiology (ESC)Developed with the special contribution of the Heart Failure Association (HFA) of the ESC. Eur Heart J.

[3] Tanai E, Frantz S (2015). Pathophysiology of heart failure. Compr Physiol.

[4] Seferovic PM, Ponikowski P, Anker SD, Bauersachs J, Chioncel O, Cleland JGF, de Boer RA, Drexel H, Ben Gal T, Hill L (2019). Clinical practice update on heart failure 2019: pharmacotherapy, procedures, devices and patient management. An expert consensus meeting report of the Heart Failure Association of the European Society of Cardiology. Eur J Heart Fail.

[5] Konstam MA, Kiernan M, Chandler A, Dhingra R, Mody FV, Eisen H, Haught WH, Wagoner L, Gupta D, Patten R (2017). Short-term effects of tolvaptan in patients with acute heart failure and volume overload. J Am Coll Cardiol.

[6] Lu T-L, Chang W-T, Chan C-H, Wu S-N (2019). Evidence for effective multiple K-current inhibitions by tolvaptan, a non-peptide antagonist of vasopressin V receptor. Front Pharmacol.

[7] Yancy CW, Jessup M, Bozkurt B, Butler J, Casey DE, Colvin MM, Drazner MH, Filippatos GS, Fonarow GC, Givertz MM (2017). 2017 ACC/AHA/HFSA Focused Update of the 2013 ACCF/AHA Guideline for the Management of Heart Failure: a Report of the American College of Cardiology/American Heart Association Task Force on Clinical Practice Guidelines and the Heart Failure Society of America. Circulation.

[8] Felker GM, Mentz RJ, Cole RT, Adams KF, Egnaczyk GF, Fiuzat M, Patel CB, Echols M, Khouri MG, Tauras JM (2017). Efficacy and safety of tolvaptan in patients hospitalized with acute heart failure. J Am Coll Cardiol.

[9] Matsue Y, Suzuki M, Torii S, Yamaguchi S, Fukamizu S, Ono Y, Fujii H, Kitai T, Nishioka T, Sugi K (2016). Clinical effectiveness of tolvaptan in patients with acute heart failure and renal dysfunction. J Card Fail.

[10] Matsue Y, Ter Maaten JM, Suzuki M, Torii S, Yamaguchi S, Fukamizu S, Ono Y, Fujii H, Kitai T, Nishioka T (2017). Early treatment with tolvaptan improves diuretic response in acute heart failure with renal dysfunction. Clin Res Cardiol.

[11] Shirakabe A, Hata N, Yamamoto M, Kobayashi N, Shinada T, Tomita K, Tsurumi M, Matsushita M, Okazaki H, Yamamoto Y (2014). Immediate administration of tolvaptan prevents the exacerbation of acute kidney injury and improves the mid-term prognosis of patients with severely decompensated acute heart failure. Circ J.

[12] Gheorghiade M, Follath F, Ponikowski P, Barsuk JH, Blair JEA, Cleland JG, Dickstein K, Drazner MH, Fonarow GC, Jaarsma T (2014). Assessing and grading congestion in acute heart failure: a scientific statement from the Acute Heart Failure Committee of the Heart Failure Association of the European Society of Cardiology and endorsed by the European Society of Intensive Care Medicine. Eur J Heart Fail.

[13] Udelson JE, Mcgrew FA, Enrique F, Hassan I, Stewart K, Gregory K, Terrence OB, Kronenberg MW, Christopher Z, Cesare O (2007). Multicenter, randomized, double-blind, placebo-controlled study on the effect of oral tolvaptan on left ventricular dilation and function in patients with heart failure and systolic dysfunction. J Am Coll Cardiol.

[14] Swedberg K, Komajda M, Böhm M, Borer JS, Ford I, Dubost-Brama A, Lerebours G, Tavazzi L (2010). Ivabradine and outcomes in chronic heart failure (SHIFT): a randomised placebo-controlled study. Lancet.

[15] Tavazzi L, Swedberg K, Komajda M, Böhm M, Borer JS, Lainscak M, Ford I (2013). Efficacy and safety of ivabradine in chronic heart failure across the age spectrum: insights from the SHIFT study. Eur J Heart Fail.

[16] Yancy CW, Jessup M, Bozkurt B, Butler J, Casey DE, Colvin MM, Drazner MH, Filippatos G, Fonarow GC, Givertz MM (2016). 2016 ACC/AHA/HFSA Focused Update on New Pharmacological Therapy for Heart Failure: an Update of the 2013 ACCF/AHA Guideline for the Management of Heart Failure: A Report of the American College of Cardiology/American Heart Association Task Force on Clinical Practice Guidelines and the Heart Failure Society of America. Circulation.

[17] Bagriy AE, Schukina EV, Samoilova OV, Pricolota OA, Malovichko SI, Pricolota AV, Bagriy EA (2015). Addition of ivabradine to β-blocker improves exercise capacity in systolic heart failure patients in a prospective, open-label study. Adv Ther.

[18] Volterrani M, Cice G, Caminiti G, Vitale C, D’Isa S, Perrone Filardi P, Acquistapace F, Marazzi G, Fini M, Rosano GMC (2011). Effect of carvedilol, ivabradine or their combination on exercise capacity in patients with Heart Failure (the CARVIVA HF trial). Int J Cardiol.

[19] McMurray JJV, Packer M, Desai AS, Gong J, Lefkowitz MP, Rizkala AR, Rouleau JL, Shi VC, Solomon SD, Swedberg K (2014). Angiotensin-neprilysin inhibition versus enalapril in heart failure. N Engl J Med.

[20] Simpson J, Jhund PS, Silva Cardoso J, Martinez F, Mosterd A, Ramires F, Rizkala AR, Senni M, Squire I, Gong J (2015). Comparing LCZ696 with enalapril according to baseline risk using the MAGGIC and EMPHASIS-HF risk scores: an analysis of mortality and morbidity in PARADIGM-HF. J Am Coll Cardiol.

[21] Lewis EF, Claggett BL, McMurray JJV, Packer M, Lefkowitz MP, Rouleau JL, Liu J, Shi VC, Zile MR, Desai AS (2017). Health-related quality of life outcomes in PARADIGM-HF. Circ Heart Fail..

[22] Vardeny O, Claggett B, Kachadourian J, Desai AS, Packer M, Rouleau J, Zile MR, Swedberg K, Lefkowitz M, Shi V (2019). Reduced loop diuretic use in patients taking sacubitril/valsartan compared with enalapril: the PARADIGM-HF trial. Eur J Heart Fail.

[23] Packer M, McMurray JJV, Desai AS, Gong J, Lefkowitz MP, Rizkala AR, Rouleau JL, Shi VC, Solomon SD, Swedberg K (2015). Angiotensin receptor neprilysin inhibition compared with enalapril on the risk of clinical progression in surviving patients with heart failure. Circulation.

[24] Velazquez EJ, Morrow DA, DeVore AD, Duffy CI, Ambrosy AP, McCague K, Rocha R, Braunwald E, Investigators P-H (2019). Angiotensin-neprilysin inhibition in acute decompensated heart failure. N Engl J Med.

[25] Januzzi JL, Prescott MF, Butler J, Felker GM, Maisel AS, McCague K, Camacho A, Piña IL, Rocha RA, Shah AM (2019). Association of change in N-Terminal Pro-B-type natriuretic peptide following initiation of sacubitril-valsartan treatment with cardiac structure and function in patients with heart failure with reduced ejection fraction. JAMA..

[26] Desai AS, Solomon SD, Shah AM, Claggett BL, Fang JC, Izzo J, McCague K, Abbas CA, Rocha R, Mitchell GF (2019). Effect of sacubitril-valsartan vs enalapril on aortic stiffness in patients with heart failure and reduced ejection fraction: a randomized clinical trial. JAMA.

[27] Wachter R, Michele S, Witte K, Straburzyńska-Migaj E, Belohlavek J, Fonseca C, Mueller C, Lonn E, Bao W, Noe A (2019). In-hospital initiation of sacubitril/valsartan in stabilised patients with heart failure and reduced ejection fraction naïve to renin-angiotensin system blocker: an analysis of the transition study. Heart Lung Circ.

[28] Wachter R, Michele S, Witte K, Straburzynska-Migaj E, Belohlavek J, Fonseca C, Mueller C, Lonn E, Bao W, Noe A et al. Initiation of sacubitril/valsartan in patients with de novo heart failure with reduced ejection fraction: an analysis of the transition study. Heart Lung Circ. 2019;28.

[29] Jhund P, Claggett B, Packer M, Zile M, Voors A, Pieske B, Lefkowitz M, Shi V, Bransford T, McMurray J (2014). The efficacy of the angiotensin receptor neprilysin inhibitor, LCZ696, in patients with heart failure with preserved ejection fraction is independent of blood pressure lowering. J Am Coll Cardiol..

[30] Jhund PS, Claggett B, Packer M, Zile MR, Voors AA, Pieske B, Lefkowitz M, Shi V, Bransford T, McMurray JJV (2014). Independence of the blood pressure lowering effect and efficacy of the angiotensin receptor neprilysin inhibitor, LCZ696, in patients with heart failure with preserved ejection fraction: an analysis of the PARAMOUNT trial. Eur J Heart Fail.

[31] Solomon SD, McMurray JJV, Anand IS, Ge J, Lam CSP, Maggioni AP, Martinez F, Packer M, Pfeffer MA, Pieske B (2019). Angiotensin-neprilysin inhibition in heart failure with preserved ejection fraction. N Engl J Med.

[32] Burke RM, Lighthouse JK, Mickelsen DM, Small EM (2019). Sacubitril/valsartan decreases cardiac fibrosis in left ventricle pressure overload by restoring PKG signaling in cardiac fibroblasts. Circ Heart Fail.

[33] Flather MD, Shibata MC, Coats AJS, Van Veldhuisen DJ, Parkhomenko A, Borbola J, Cohen-Solal A, Dumitrascu D, Ferrari R, Lechat P (2005). Randomized trial to determine the effect of nebivolol on mortality and cardiovascular hospital admission in elderly patients with heart failure (SENIORS). Eur Heart J.

[34] van Veldhuisen DJ, Cohen-Solal A, Böhm M, Anker SD, Babalis D, Roughton M, Coats AJS, Poole-Wilson PA, Flather MD (2009). Beta-blockade with nebivolol in elderly heart failure patients with impaired and preserved left ventricular ejection fraction: data From SENIORS (Study of Effects of Nebivolol Intervention on Outcomes and Rehospitalization in Seniors With Heart Failure). J Am Coll Cardiol.

[35] Montero-Perez-Barquero M, Flather M, Roughton M, Coats A, Böhm M, Van Veldhuisen DJ, Babalis D, Solal AC, Manzano L (2014). Influence of systolic blood pressure on clinical outcomes in elderly heart failure patients treated with nebivolol: data from the SENIORS trial. Eur J Heart Fail.

[36] de Boer RA, Doehner W, van der Horst ICC, Anker SD, Babalis D, Roughton M, Coats AJ, Flather MD, van Veldhuisen DJ (2010). Influence of diabetes mellitus and hyperglycemia on prognosis in patients > or =70 years old with heart failure and effects of nebivolol (data from the Study of Effects of Nebivolol Intervention on Outcomes and Rehospitalization in Seniors with heart failure [SENIORS]). Am J Cardiol..

[37] Dobre D, van Veldhuisen DJ, Mordenti G, Vintila M, Haaijer-Ruskamp FM, Coats AJS, Poole-Wilson PA, Flather MD (2007). Tolerability and dose-related effects of nebivolol in elderly patients with heart failure: data from the Study of the Effects of Nebivolol Intervention on Outcomes and Rehospitalisation in Seniors with Heart Failure (SENIORS) trial. Am Heart J.

[38] Sessa M, Mascolo A, Mortensen RN, Andersen MP, Rosano GMC, Capuano A, Rossi F, Gislason G, Enghusen-Poulsen H, Torp-Pedersen C (2018). Relationship between heart failure, concurrent chronic obstructive pulmonary disease and beta-blocker use: a Danish nationwide cohort study. Eur J Heart Fail.

[39] Brehm BR, Wolf SC, Görner S, Buck-Müller N, Risler T (2002). Effect of nebivolol on left ventricular function in patients with chronic heart failure: a pilot study. Eur J Heart Fail.

[40] Conraads VM, Metra M, Kamp O, De Keulenaer GW, Pieske B, Zamorano J, Vardas PE, Böhm M, Dei Cas L (2012). Effects of the long-term administration of nebivolol on the clinical symptoms, exercise capacity, and left ventricular function of patients with diastolic dysfunction: results of the ELANDD study. Eur J Heart Fail.

[41] Contini M, Apostolo A, Cattadori G, Paolillo S, Iorio A, Bertella E, Salvioni E, Alimento M, Farina S, Palermo P (2013). Multiparametric comparison of CARvedilol, vs. NEbivolol, vs. BIsoprolol in moderate heart failure: the CARNEBI trial. Int J Cardiol..

[42] Follath F, Cleland JGF, Just H, Papp JGY, Scholz H, Peuhkurinen K, Harjola VP, Mitrovic V, Abdalla M, Sandell EP (2002). Efficacy and safety of intravenous levosimendan compared with dobutamine in severe low-output heart failure (the LIDO study): a randomised double-blind trial. Lancet.

[43] Mebazaa A, Nieminen MS, Packer M, Cohen-Solal A, Kleber FX, Pocock SJ, Thakkar R, Padley RJ, Põder P, Kivikko M (2007). Levosimendan vs dobutamine for patients with acute decompensated heart failure: the SURVIVE randomized trial. JAMA.

[44] Packer M, Colucci W, Fisher L, Massie BM, Teerlink JR, Young J, Padley RJ, Thakkar R, Delgado-Herrera L, Salon J (2013). Effect of levosimendan on the short-term clinical course of patients with acutely decompensated heart failure. JACC Heart Fail.

[45] Altenberger J, Parissis JT, Costard-Jaeckle A, Winter A, Ebner C, Karavidas A, Sihorsch K, Avgeropoulou E, Weber T, Dimopoulos L (2014). Efficacy and safety of the pulsed infusions of levosimendan in outpatients with advanced heart failure (LevoRep) study: a multicentre randomized trial. Eur J Heart Fail.

[46] Zhang D, Yao Y, Qian J, Huang J (2015). Levosimendan improves clinical outcomes of refractory heart failure in elderly Chinese patients. Med Sci Monit.

[47] Comín-Colet J, Manito N, Segovia-Cubero J, Delgado J, García Pinilla JM, Almenar L, Crespo-Leiro MG, Sionis A, Blasco T, Pascual-Figal D (2018). Efficacy and safety of intermittent intravenous outpatient administration of levosimendan in patients with advanced heart failure: the LION-HEART multicentre randomised trial. Eur J Heart Fail.

[48] Husebye T, Eritsland J, Müller C, Sandvik L, Arnesen H, Seljeflot I, Mangschau A, Bjørnerheim R, Andersen GØ (2013). Levosimendan in acute heart failure following primary percutaneous coronary intervention-treated acute ST-elevation myocardial infarction. Results from the LEAF trial: a randomized, placebo-controlled study. Eur J Heart Fail..

[49] Jia Z, Guo M, Zhang Y-Q, Liang H-Q, Zhang L-Y, Song Y (2014). Efficacy of intravenous levosimendan in patients with heart failure complicated by acute myocardial infarction. Cardiology.

[50] García-González MJ, de Mora-Martín M, López-Fernández S, López-Díaz J, Martínez-Sellés M, Romero-García J, Cordero M, Lara-Padrón A, Marrero-Rodríguez F, del Mar García-Saiz M (2013). Rationale and design of a randomized, double-blind, placebo controlled multicenter trial to study efficacy, security, and long term effects of intermittent repeated levosimendan administration in patients with advanced heart failure: LAICA study. Cardiovasc Drugs Ther.

[51] Pölzl G, Allipour Birgani S, Comín-Colet J, Delgado JF, Fedele F, García-Gonzáles MJ, Gustafsson F, Masip J, Papp Z, Störk S (2019). Repetitive levosimendan infusions for patients with advanced chronic heart failure in the vulnerable post-discharge period. ESC Heart Fail.

[52] Eschalier R, McMurray JJV, Swedberg K, van Veldhuisen DJ, Krum H, Pocock SJ, Shi H, Vincent J, Rossignol P, Zannad F (2013). Safety and efficacy of eplerenone in patients at high risk for hyperkalemia and/or worsening renal function: analyses of the EMPHASIS-HF study subgroups (Eplerenone in Mild Patients Hospitalization And SurvIval Study in Heart Failure). J Am Coll Cardiol.

[53] Girerd N, Collier T, Pocock S, Krum H, McMurray JJ, Swedberg K, Van Veldhuisen DJ, Vincent J, Pitt B, Zannad F (2015). Clinical benefits of eplerenone in patients with systolic heart failure and mild symptoms when initiated shortly after hospital discharge: analysis from the EMPHASIS-HF trial. Eur Heart J.

[54] Rossignol P, Dobre D, McMurray JJV, Swedberg K, Krum H, van Veldhuisen DJ, Shi H, Messig M, Vincent J, Girerd N (2014). Incidence, determinants, and prognostic significance of hyperkalemia and worsening renal function in patients with heart failure receiving the mineralocorticoid receptor antagonist eplerenone or placebo in addition to optimal medical therapy: results from the Eplerenone in Mild Patients Hospitalization and Survival Study in Heart Failure (EMPHASIS-HF). Circ Heart Fail.

[55] Ferreira JP, Abreu P, McMurray JJV, van Veldhuisen DJ, Swedberg K, Pocock SJ, Vincent J, Lins K, Rossignol P, Pitt B (2019). Renal function stratified dose comparisons of eplerenone versus placebo in the EMPHASIS-HF trial. Eur J Heart Fail.

[56] Cannon JA, Collier TJ, Shen L, Swedberg K, Krum H, Van Veldhuisen DJ, Vincent J, Pocock SJ, Pitt B, Zannad F (2015). Clinical outcomes according to QRS duration and morphology in the Eplerenone in Mild Patients: hospitalization and SurvIval Study in Heart Failure (EMPHASIS-HF). Eur J Heart Fail.

[57] Chin KL, Collier T, Pocock S, Pitt B, McMurray JJV, van Veldhuisen DJ, Swedberg K, Vincent J, Zannad F, Liew D (2019). Impact of eplerenone on major cardiovascular outcomes in patients with systolic heart failure according to baseline heart rate. Clin Res Cardiol.

[58] Rossignol P, Girerd N, Bakris G, Vardeny O, Claggett B, McMurray JJV, Swedberg K, Krum H, van Veldhuisen DJ, Shi H (2017). Impact of eplerenone on cardiovascular outcomes in heart failure patients with hypokalaemia. Eur J Heart Fail.

[59] Olivier A, Pitt B, Girerd N, Lamiral Z, Machu J-L, McMurray JJV, Swedberg K, van Veldhuisen DJ, Collier TJ, Pocock SJ (2017). Effect of eplerenone in patients with heart failure and reduced ejection fraction: potential effect modification by abdominal obesity. Insight from the EMPHASIS-HF trial. Eur J Heart Fail..

[60] Tsutsui H, Ito H, Kitakaze M, Komuro I, Murohara T, Izumi T, Sunagawa K, Yasumura Y, Yano M, Yamamoto K (2017). Double-blind, randomized, placebo-controlled trial evaluating the efficacy and safety of eplerenone in Japanese patients with chronic heart failure (J-EMPHASIS-HF). Circ J.

[61] Ferreira JP, Rossello X, Pitt B, Rossignol P, Zannad F (2019). Eplerenone in patients with myocardial infarction and “mid-range” ejection fraction: an analysis from the EPHESUS trial. Clin Cardiol.

[62] Iqbal J, Fay R, Adlam D, Squire I, Parviz Y, Gunn J, Pitt B, Zannad F (2014). Effect of eplerenone in percutaneous coronary intervention-treated post-myocardial infarction patients with left ventricular systolic dysfunction: a subanalysis of the EPHESUS trial. Eur J Heart Fail.

[63] Korol S, White M, O’Meara E, Tournoux F, Racine N, Ducharme A, Rouleau J-L, Liszkowski M, Mansour A, Jutras M (2018). A comparison of the effects of selective and non-selective mineralocorticoid antagonism on glucose homeostasis of heart failure patients with glucose intolerance or type II diabetes: a randomized controlled double-blind trial. Am Heart J.

[64] Filippatos G, Anker SD, Böhm M, Gheorghiade M, Køber L, Krum H, Maggioni AP, Ponikowski P, Voors AA, Zannad F (2016). A randomized controlled study of finerenone vs. eplerenone in patients with worsening chronic heart failure and diabetes mellitus and/or chronic kidney disease. Eur Heart J..

[65] Sato N, Ajioka M, Yamada T, Kato M, Myoishi M, Yamada T, Kim S-Y, Nowack C, Kolkhof P, Shiga T (2016). A randomized controlled study of finerenone vs. eplerenone in Japanese patients with worsening chronic heart failure and diabetes and/or chronic kidney disease. Circ J..

[66] Pitt B, Pfeffer MA, Assmann SF, Boineau R, Anand IS, Claggett B, Clausell N, Desai AS, Diaz R, Fleg JL (2014). Spironolactone for heart failure with preserved ejection fraction. N Engl J Med.

[67] Pfeffer MA, Claggett B, Assmann SF, Boineau R, Anand IS, Clausell N, Desai AS, Diaz R, Fleg JL, Gordeev I (2015). Regional variation in patients and outcomes in the Treatment of Preserved Cardiac Function Heart Failure With an Aldosterone Antagonist (TOPCAT) trial. Circulation.

[68] Solomon SD, Claggett B, Lewis EF, Desai A, Anand I, Sweitzer NK, O’Meara E, Shah SJ, McKinlay S, Fleg JL (2016). Influence of ejection fraction on outcomes and efficacy of spironolactone in patients with heart failure with preserved ejection fraction. Eur Heart J.

[69] Shah AM, Claggett B, Sweitzer NK, Shah SJ, Deswal A, Anand IS, Fleg JL, Pitt B, Pfeffer MA, Solomon SD (2015). Prognostic importance of changes in cardiac structure and function in heart failure with preserved ejection fraction and the impact of spironolactone. Circulation Heart failure.

[70] Edelmann F, Wachter R, Schmidt AG, Kraigher-Krainer E, Colantonio C, Kamke W, Duvinage A, Stahrenberg R, Durstewitz K, Löffler M (2013). Effect of spironolactone on diastolic function and exercise capacity in patients with heart failure with preserved ejection fraction: the Aldo-DHF randomized controlled trial. JAMA.

[71] Ravassa S, Trippel T, Bach D, Bachran D, González A, López B, Wachter R, Hasenfuss G, Delles C, Dominiczak AF (2018). Biomarker-based phenotyping of myocardial fibrosis identifies patients with heart failure with preserved ejection fraction resistant to the beneficial effects of spironolactone: results from the Aldo-DHF trial. Eur J Heart Fail.

[72] Upadhya B, Hundley WG, Brubaker PH, Morgan TM, Stewart KP, Kitzman DW (2017). Effect of spironolactone on exercise tolerance and arterial function in older adults with heart failure with preserved ejection fraction. J Am Geriatr Soc.

[73] Kosmala W, Przewlocka-Kosmala M, Marwick TH (2019). Association of active and passive components of LV diastolic filling with exercise intolerance in heart failure with preserved ejection fraction: mechanistic insights from spironolactone response. JACC Cardiovasc Imaging.

[74] Butler J, Anstrom KJ, Felker GM, Givertz MM, Kalogeropoulos AP, Konstam MA, Mann DL, Margulies KB, McNulty SE, Mentz RJ (2017). Efficacy and safety of spironolactone in acute heart failure: The ATHENA-HF randomized clinical trial. JAMA Cardiol.

[75] Pitt B, Remme W, Zannad F, Neaton J, Martinez F, Roniker B, Bittman R, Hurley S, Kleiman J, Gatlin M (2003). Eplerenone, a selective aldosterone blocker, in patients with left ventricular dysfunction after myocardial infarction. N Engl J Med.

[76] Asakura M, Yamamoto H, Asai K, Hanatani A, Hirata K-I, Hirayakma A, Kimura K, Kobayashi Y, Momomura S-I, Nakagawa Y (2015). Rationale and design of the double-blind, randomized, placebo-controlled multicenter trial on efficacy of Early Initiation of Eplerenone Treatment in Patients with Acute Heart Failure (EARLIER). Cardiovasc Drugs Ther..

[77] Rørth R, Dewan P, Kristensen SL, Jhund PS, Petrie MC, Køber L, McMurray JJV (2019). Efficacy of an implantable cardioverter-defibrillator in patients with diabetes and heart failure and reduced ejection fraction. Clin Res Cardiol.

[78] Køber L, Thune JJ, Nielsen JC, Haarbo J, Videbæk L, Korup E, Jensen G, Hildebrandt P, Steffensen FH, Bruun NE (2016). Defibrillator implantation in patients with nonischemic systolic heart failure. N Engl J Med.

[79] Shen L, Jhund PS, Petrie MC, Claggett BL, Barlera S, Cleland JGF, Dargie HJ, Granger CB, Kjekshus J, Køber L (2017). Declining risk of sudden death in heart failure. N Engl J Med.

[80] Vaduganathan M, Claggett BL, Chatterjee NA, Anand IS, Sweitzer NK, Fang JC, O’Meara E, Shah SJ, Hegde SM, Desai AS (2018). Sudden death in heart failure with preserved ejection fraction: a competing risks analysis from the TOPCAT trial. JACC Heart Fail.

[81] Manolis AS, Manolis AA, Manolis TA, Melita H (2019). Sudden death in heart failure with preserved ejection fraction and beyond: an elusive target. Heart Fail Rev.

[82] Sohaib SMA, Finegold JA, Nijjer SS, Hossain R, Linde C, Levy WC, Sutton R, Kanagaratnam P, Francis DP, Whinnett ZI (2015). Opportunity to increase life span in narrow QRS cardiac resynchronization therapy recipients by deactivating ventricular pacing: evidence from randomized controlled trials. JACC Heart Fail.

[83] Cleland JG, Abraham WT, Linde C, Gold MR, Young JB, Claude Daubert J, Sherfesee L, Wells GA, Tang ASL (2013). An individual patient meta-analysis of five randomized trials assessing the effects of cardiac resynchronization therapy on morbidity and mortality in patients with symptomatic heart failure. Eur Heart J.

[84] Tang ASL, Wells GA, Talajic M, Arnold MO, Sheldon R, Connolly S, Hohnloser SH, Nichol G, Birnie DH, Sapp JL (2010). Cardiac-resynchronization therapy for mild-to-moderate heart failure. N Engl J Med.

[85] Goldenberg I, Kutyifa V, Klein HU, Cannom DS, Brown MW, Dan A, Daubert JP, Estes NAM, Foster E, Greenberg H (2014). Survival with cardiac-resynchronization therapy in mild heart failure. N Engl J Med.

[86] Linde C, Gold MR, Abraham WT, John Sutton M, Ghio S, Cerkvenik J, Daubert C (2013). Long-term impact of cardiac resynchronization therapy in mild heart failure: 5-year results from the REsynchronization reVErses Remodeling in Systolic left vEntricular dysfunction (REVERSE) study. Eur Heart J.

[87] Curtis AB, Worley SJ, Adamson PB, Chung ES, Niazi I, Sherfesee L, Shinn T, Sutton MSJ (2013). Biventricular pacing for atrioventricular block and systolic dysfunction. N Engl J Med.

[88] Abraham WT, Kuck K-H, Goldsmith RL, Lindenfeld J, Reddy VY, Carson PE, Mann DL, Saville B, Parise H, Chan R (2018). A randomized controlled trial to evaluate the safety and efficacy of cardiac contractility modulation. JACC Heart Fail.

[89] Kadish A, Nademanee K, Volosin K, Krueger S, Neelagaru S, Raval N, Obel O, Weiner S, Wish M, Carson P (2011). A randomized controlled trial evaluating the safety and efficacy of cardiac contractility modulation in advanced heart failure. Am Heart J.

[90] Borggrefe MM, Lawo T, Butter C, Schmidinger H, Lunati M, Pieske B, Misier AR, Curnis A, Böcker D, Remppis A (2008). Randomized, double blind study of non-excitatory, cardiac contractility modulation electrical impulses for symptomatic heart failure. Eur Heart J.

[91] Witte K, Hasenfuss G, Kloppe A, Burkhoff D, Green M, Moss J, Peel A, Mealing S, Durand Zaleski I, Cowie MR (2019). Cost-effectiveness of a cardiac contractility modulation device in heart failure with normal QRS duration. ESC Heart Fail.

[92] Zannad F, De Ferrari GM, Tuinenburg AE, Wright D, Brugada J, Butter C, Klein H, Stolen C, Meyer S, Stein KM (2015). Chronic vagal stimulation for the treatment of low ejection fraction heart failure: results of the NEural Cardiac TherApy foR Heart Failure (NECTAR-HF) randomized controlled trial. Eur Heart J.

[93] De Ferrari GM, Stolen C, Tuinenburg AE, Wright DJ, Brugada J, Butter C, Klein H, Neuzil P, Botman C, Castel MA (2017). Long-term vagal stimulation for heart failure: eighteen month results from the NEural Cardiac TherApy foR Heart Failure (NECTAR-HF) trial. Int J Cardiol.

[94] Premchand RK, Sharma K, Mittal S, Monteiro R, Dixit S, Libbus I, DiCarlo LA, Ardell JL, Rector TS, Amurthur B (2014). Autonomic regulation therapy via left or right cervical vagus nerve stimulation in patients with chronic heart failure: results of the ANTHEM-HF trial. J Card Fail.

[95] Premchand RK, Sharma K, Mittal S, Monteiro R, Dixit S, Libbus I, DiCarlo LA, Ardell JL, Rector TS, Amurthur B (2016). Extended follow-up of patients with heart failure receiving autonomic regulation therapy in the ANTHEM-HF study. J Card Fail.

[96] Gold MR, Van Veldhuisen DJ, Hauptman PJ, Borggrefe M, Kubo SH, Lieberman RA, Milasinovic G, Berman BJ, Djordjevic S, Neelagaru S (2016). Vagus nerve stimulation for the treatment of heart failure: The INOVATE-HF trial. J Am Coll Cardiol.

[97] Trivedi JR, Cheng A, Singh R, Williams ML, Slaughter MS (2014). Survival on the heart transplant waiting list: impact of continuous flow left ventricular assist device as bridge to transplant. Ann Thorac Surg.

[98] Stewart GC, Givertz MM (2012). Mechanical circulatory support for advanced heart failure: patients and technology in evolution. Circulation.

[99] Estep JD, Starling RC, Horstmanshof DA, Milano CA, Selzman CH, Shah KB, Loebe M, Moazami N, Long JW, Stehlik J (2015). Risk assessment and comparative effectiveness of left ventricular assist device and medical management in ambulatory heart failure patients: results from the ROADMAP study. J Am Coll Cardiol.

[100] Riebandt J, Haberl T, Mahr S, Laufer G, Rajek A, Steinlechner B, Schima H, Zimpfer D (2014). Preoperative patient optimization using extracorporeal life support improves outcomes of INTERMACS Level I patients receiving a permanent ventricular assist device. Eur J Card Thorac Surg..

[101] Abraham WT, Zile MR, Weaver FA, Butter C, Ducharme A, Halbach M, Klug D, Lovett EG, Müller-Ehmsen J, Schafer JE (2015). Baroreflex activation therapy for the treatment of heart failure with a reduced ejection fraction. JACC Heart Fail.

[102] Zile MR, Abraham WT, Weaver FA, Butter C, Ducharme A, Halbach M, Klug D, Lovett EG, Müller-Ehmsen J, Schafer JE (2015). Baroreflex activation therapy for the treatment of heart failure with a reduced ejection fraction: safety and efficacy in patients with and without cardiac resynchronization therapy. Eur J Heart Fail.

[103] Halbach M, Abraham WT, Butter C, Ducharme A, Klug D, Little WC, Reuter H, Schafer JE, Senni M, Swarup V (2018). Baroreflex activation therapy for the treatment of heart failure with reduced ejection fraction in patients with and without coronary artery disease. Int J Cardiol.

[104] Dell’Oro R, Gronda E, Seravalle G, Costantino G, Alberti L, Baronio B, Staine T, Vanoli E, Mancia G, Grassi G (2017). Restoration of normal sympathetic neural function in heart failure following baroreflex activation therapy: final 43-month study report. J Hypertens.

[105] Seferović PM, Coats AJS, Ponikowski P, Filippatos G, Huelsmann M, Jhund PS, Polovina MM, Komajda M, Seferović J, Sari I (2020). European Society of Cardiology/Heart Failure Association position paper on the role and safety of new glucose-lowering drugs in patients with heart failure. Eur J Heart Fail.

[106] McHugh K, DeVore AD, Wu J, Matsouaka RA, Fonarow GC, Heidenreich PA, Yancy CW, Green JB, Altman N, Hernandez AF (2019). Heart failure with preserved ejection fraction and diabetes: JACC state-of-the-art review. J Am Coll Cardiol.

[107] Udell JA, Yuan Z, Rush T, Sicignano NM, Galitz M, Rosenthal N (2018). Cardiovascular outcomes and risks after initiation of a sodium glucose cotransporter 2 inhibitor: results from the EASEL Population-Based Cohort Study (Evidence for Cardiovascular Outcomes With Sodium Glucose Cotransporter 2 Inhibitors in the Real World). Circulation.

[108] Kosiborod M, Lam CSP, Kohsaka S, Kim DJ, Karasik A, Shaw J, Tangri N, Goh S-Y, Thuresson M, Chen H (2018). Cardiovascular events associated with SGLT-2 inhibitors versus other glucose-lowering drugs: The CVD-REAL 2 study. J Am Coll Cardiol.

[109] Kosiborod M, Cavender MA, Fu AZ, Wilding JP, Khunti K, Holl RW, Norhammar A, Birkeland KI, Jørgensen ME, Thuresson M (2017). Lower risk of heart failure and death in patients initiated on sodium-glucose cotransporter-2 inhibitors versus other glucose-lowering Drugs: The CVD-REAL Study (Comparative Effectiveness of Cardiovascular Outcomes in New Users of Sodium-Glucose Cotransporter-2 Inhibitors). Circulation.

[110] Pasternak B, Ueda P, Eliasson B, Svensson A-M, Franzén S, Gudbjörnsdottir S, Hveem K, Jonasson C, Wintzell V, Melbye M (2019). Use of sodium glucose cotransporter 2 inhibitors and risk of major cardiovascular events and heart failure: Scandinavian register based cohort study. BMJ.

[111] Schork A, Saynisch J, Vosseler A, Jaghutriz BA, Heyne N, Peter A, Häring H-U, Stefan N, Fritsche A, Artunc F (2019). Effect of SGLT2 inhibitors on body composition, fluid status and renin-angiotensin-aldosterone system in type 2 diabetes: a prospective study using bioimpedance spectroscopy. Cardiovasc Diabetol.

[112] Zinman B, Wanner C, Lachin JM, Fitchett D, Bluhmki E, Hantel S, Mattheus M, Devins T, Johansen OE, Woerle HJ (2015). Empagliflozin, cardiovascular outcomes, and mortality in type 2 diabetes. N Engl J Med.

[113] Schernthaner G, Karasik A, Abraitienė A, Ametov AS, Gaàl Z, Gumprecht J, Janež A, Kaser S, Lalić K, Mankovsky BN (2019). Evidence from routine clinical practice: EMPRISE provides a new perspective on CVOTs. Cardiovasc Diabetol.

[114] Fitchett D, Inzucchi SE, Cannon CP, McGuire DK, Scirica BM, Johansen OE, Sambevski S, Kaspers S, Pfarr E, George JT (2019). Empagliflozin reduced mortality and hospitalization for heart failure across the spectrum of cardiovascular risk in the EMPA-REG OUTCOME trial. Circulation.

[115] Pabel S, Wagner S, Bollenberg H, Bengel P, Kovács Á, Schach C, Tirilomis P, Mustroph J, Renner A, Gummert J (2018). Empagliflozin directly improves diastolic function in human heart failure. Eur J Heart Fail.

[116] Lee H-C, Shiou Y-L, Jhuo S-J, Chang C-Y, Liu P-L, Jhuang W-J, Dai Z-K, Chen W-Y, Chen Y-F, Lee A-S (2019). The sodium-glucose co-transporter 2 inhibitor empagliflozin attenuates cardiac fibrosis and improves ventricular hemodynamics in hypertensive heart failure rats. Cardiovasc Diabetol.

[117] Norhammar A, Bodegård J, Nyström T, Thuresson M, Nathanson D, Eriksson JW (2019). Dapagliflozin and cardiovascular mortality and disease outcomes in a population with type 2 diabetes similar to that of the DECLARE-TIMI 58 trial: a nationwide observational study. Diabetes Obes Metab.

[118] McMurray JJV, DeMets DL, Inzucchi SE, Køber L, Kosiborod MN, Langkilde AM, Martinez FA, Bengtsson O, Ponikowski P, Sabatine MS (2019). A trial to evaluate the effect of the sodium-glucose co-transporter 2 inhibitor dapagliflozin on morbidity and mortality in patients with heart failure and reduced left ventricular ejection fraction (DAPA-HF). Eur J Heart Fail.

[119] McMurray JJV, Solomon SD, Inzucchi SE, Køber L, Kosiborod MN, Martinez FA, Ponikowski P, Sabatine MS, Anand IS, Bělohlávek J (2019). Dapagliflozin in patients with heart failure and reduced ejection fraction. N Engl J Med.

[120] Carbone S, Dixon DL (2019). The CANVAS Program: implications of canagliflozin on reducing cardiovascular risk in patients with type 2 diabetes mellitus. Cardiovasc Diabetol.

[121] Seferović PM, Petrie MC, Filippatos GS, Anker SD, Rosano G, Bauersachs J, Paulus WJ, Komajda M, Cosentino F, de Boer RA (2018). Type 2 diabetes mellitus and heart failure: a position statement from the Heart Failure Association of the European Society of Cardiology. Eur J Heart Fail.

[122] Wanner C, Lachin JM, Inzucchi SE, Fitchett D, Mattheus M, George J, Woerle HJ, Broedl UC, von Eynatten M, Zinman B (2018). Empagliflozin and clinical outcomes in patients with type 2 diabetes mellitus, established cardiovascular disease, and chronic kidney disease. Circulation.

[123] Liang B, Zhao Y-X, Gu N (2020). Empagliflozin improves cardiac function in heart failure with reduced ejection fraction independent of loading conditions. Cardiovasc Diabetol.

[124] Patorno E, Pawar A, Franklin JM, Najafzadeh M, Déruaz-Luyet A, Brodovicz KG, Sambevski S, Bessette LG, Santiago Ortiz AJ, Kulldorff M (2019). Empagliflozin and the risk of heart failure hospitalization in routine clinical care. Circulation.

[125] Wiviott SD, Raz I, Bonaca MP, Mosenzon O, Kato ET, Cahn A, Silverman MG, Zelniker TA, Kuder JF, Murphy SA (2019). Dapagliflozin and cardiovascular outcomes in type 2 diabetes. N Engl J Med.

[126] Furtado RHM, Bonaca MP, Raz I, Zelniker TA, Mosenzon O, Cahn A, Kuder J, Murphy SA, Bhatt DL, Leiter LA (2019). Dapagliflozin and cardiovascular outcomes in patients with type 2 diabetes mellitus and previous myocardial infarction. Circulation.

[127] Kato ET, Silverman MG, Mosenzon O, Zelniker TA, Cahn A, Furtado RHM, Kuder J, Murphy SA, Bhatt DL, Leiter LA (2019). Effect of dapagliflozin on heart failure and mortality in type 2 diabetes mellitus. Circulation.

[128] Soga F, Tanaka H, Tatsumi K, Mochizuki Y, Sano H, Toki H, Matsumoto K, Shite J, Takaoka H, Doi T (2018). Impact of dapagliflozin on left ventricular diastolic function of patients with type 2 diabetic mellitus with chronic heart failure. Cardiovasc Diabetol.

[129] Perkovic V, Jardine MJ, Neal B, Bompoint S, Heerspink HJL, Charytan DM, Edwards R, Agarwal R, Bakris G, Bull S (2019). Canagliflozin and renal outcomes in type 2 diabetes and nephropathy. N Engl J Med.

[130] Mahaffey KW, Jardine MJ, Bompoint S, Cannon CP, Neal B, Heerspink HJL, Charytan DM, Edwards R, Agarwal R, Bakris G (2019). Canagliflozin and cardiovascular and renal outcomes in type 2 diabetes mellitus and chronic kidney disease in primary and secondary cardiovascular prevention groups. Circulation.

[131] Mahaffey KW, Neal B, Perkovic V, de Zeeuw D, Fulcher G, Erondu N, Shaw W, Fabbrini E, Sun T, Li Q (2018). Canagliflozin for Primary and secondary prevention of cardiovascular events: results from the CANVAS program (Canagliflozin Cardiovascular Assessment Study). Circulation.

[132] Rådholm K, Figtree G, Perkovic V, Solomon SD, Mahaffey KW, de Zeeuw D, Fulcher G, Barrett TD, Shaw W, Desai M (2018). Canagliflozin and heart failure in type 2 diabetes mellitus. Circulation.

[133] Nikolaidis LA, Sturzu A, Stolarski C, Elahi D, Shen Y-T, Shannon RP (2004). The development of myocardial insulin resistance in conscious dogs with advanced dilated cardiomyopathy. Cardiovasc Res.

[134] Komajda M (2017). Liraglutide in heart failure: caution is needed. Eur J Heart Fail.

[135] Clarke SJ, McCormick LM, Dutka DP (2014). Optimising cardioprotection during myocardial ischaemia: targeting potential intracellular pathways with glucagon-like peptide-1. Cardiovasc Diabetol.

[136] Giblett JP, Axell RG, White PA, Clarke SJ, McCormick L, Read PA, Reinhold J, Brown AJ, O’Sullivan M, West NEJ (2016). Glucagon-like peptide-1 derived cardioprotection does not utilize a KATP-channel dependent pathway: mechanistic insights from human supply and demand ischemia studies. Cardiovasc Diabetol.

[137] McCormick LM, Heck PM, Ring LS, Kydd AC, Clarke SJ, Hoole SP, Dutka DP (2015). Glucagon-like peptide-1 protects against ischemic left ventricular dysfunction during hyperglycemia in patients with coronary artery disease and type 2 diabetes mellitus. Cardiovasc Diabetol.

[138] Sokos GG, Nikolaidis LA, Mankad S, Elahi D, Shannon RP (2006). Glucagon-like peptide-1 infusion improves left ventricular ejection fraction and functional status in patients with chronic heart failure. J Card Fail.

[139] Velez M, Peterson EL, Wells K, Swadia T, Sabbah HN, Williams LK, Lanfear DE (2015). Association of antidiabetic medications targeting the glucagon-like peptide 1 pathway and heart failure events in patients with diabetes. J Card Fail.

[140] Arturi F, Succurro E, Miceli S, Cloro C, Ruffo M, Maio R, Perticone M, Sesti G, Perticone F (2017). Liraglutide improves cardiac function in patients with type 2 diabetes and chronic heart failure. Endocrine.

[141] Marso SP, Daniels GH, Brown-Frandsen K, Kristensen P, Mann JFE, Nauck MA, Nissen SE, Pocock S, Poulter NR, Ravn LS (2016). Liraglutide and cardiovascular outcomes in type 2 diabetes. N Engl J Med.

[142] Jorsal A, Kistorp C, Holmager P, Tougaard RS, Nielsen R, Hänselmann A, Nilsson B, Møller JE, Hjort J, Rasmussen J (2017). Effect of liraglutide, a glucagon-like peptide-1 analogue, on left ventricular function in stable chronic heart failure patients with and without diabetes (LIVE)-a multicentre, double-blind, randomised, placebo-controlled trial. Eur J Heart Fail.

[143] Margulies KB, Hernandez AF, Redfield MM, Givertz MM, Oliveira GH, Cole R, Mann DL, Whellan DJ, Kiernan MS, Felker GM (2016). Effects of liraglutide on clinical stability among patients with advanced heart failure and reduced ejection fraction: a randomized clinical trial. JAMA.

[144] Marso SP, Bain SC, Consoli A, Eliaschewitz FG, Jódar E, Leiter LA, Lingvay I, Rosenstock J, Seufert J, Warren ML (2016). Semaglutide and cardiovascular outcomes in patients with type 2 diabetes. N Engl J Med.

[145] Aroda VR, Ahmann A, Cariou B, Chow F, Davies MJ, Jódar E, Mehta R, Woo V, Lingvay I (2019). Comparative efficacy, safety, and cardiovascular outcomes with once-weekly subcutaneous semaglutide in the treatment of type 2 diabetes: insights from the SUSTAIN 1–7 trials. Diabetes Metab.

[146] Green JB, Bethel MA, Armstrong PW, Buse JB, Engel SS, Garg J, Josse R, Kaufman KD, Koglin J, Korn S (2015). Effect of sitagliptin on cardiovascular outcomes in type 2 diabetes. N Engl J Med.

[147] McGuire DK, Van de Werf F, Armstrong PW, Standl E, Koglin J, Green JB, Bethel MA, Cornel JH, Lopes RD, Halvorsen S (2016). Association between sitagliptin use and heart failure hospitalization and related outcomes in type 2 diabetes mellitus: secondary analysis of a randomized clinical trial. JAMA Cardiol.

[148] Scirica BM, Bhatt DL, Braunwald E, Steg PG, Davidson J, Hirshberg B, Ohman P, Frederich R, Wiviott SD, Hoffman EB (2013). Saxagliptin and cardiovascular outcomes in patients with type 2 diabetes mellitus. N Engl J Med.

[149] McMurray JJV, Ponikowski P, Bolli GB, Lukashevich V, Kozlovski P, Kothny W, Lewsey JD, Krum H (2018). Effects of vildagliptin on ventricular function in patients with type 2 diabetes mellitus and heart failure: a randomized placebo-controlled trial. JACC Heart Fail..

[150] McGuire DK, Alexander JH, Johansen OE, Perkovic V, Rosenstock J, Cooper ME, Wanner C, Kahn SE, Toto RD, Zinman B (2019). Linagliptin effects on heart failure and related outcomes in individuals with type 2 diabetes mellitus at high cardiovascular and renal risk in CARMELINA. Circulation.

[151] Gantz I, Chen M, Suryawanshi S, Ntabadde C, Shah S, O’Neill EA, Engel SS, Kaufman KD, Lai E (2017). A randomized, placebo-controlled study of the cardiovascular safety of the once-weekly DPP-4 inhibitor omarigliptin in patients with type 2 diabetes mellitus. Cardiovasc Diabetol.

[152] Fadini GP, Bonora BM, Albiero M, Zaninotto M, Plebani M, Avogaro A (2017). DPP-4 inhibition has no acute effect on BNP and its N-terminal pro-hormone measured by commercial immune-assays. A randomized cross-over trial in patients with type 2 diabetes. Cardiovasc Diabetol.

[153] Jarolim P, White WB, Cannon CP, Gao Q, Morrow DA (2018). Serial measurement of natriuretic peptides and cardiovascular outcomes in patients with type 2 diabetes in the EXAMINE trial. Diabetes Care.

[154] Ling L-H, Kistler PM, Kalman JM, Schilling RJ, Hunter RJ (2016). Comorbidity of atrial fibrillation and heart failure. Nat Rev Cardiol.

[155] Luo N, Merrill P, Parikh KS, Whellan DJ, Piña IL, Fiuzat M, Kraus WE, Kitzman DW, Keteyian SJ, O’Connor CM (2017). Exercise training in patients with chronic heart failure and atrial fibrillation. J Am Coll Cardiol.

[156] Mogensen UM, Jhund PS, Abraham WT, Desai AS, Dickstein K, Packer M, Rouleau JL, Solomon SD, Swedberg K, Zile MR (2017). Type of atrial fibrillation and outcomes in patients with heart failure and reduced ejection fraction. J Am Coll Cardiol.

[157] Cikes M, Claggett B, Shah AM, Desai AS, Lewis EF, Shah SJ, Anand IS, O’Meara E, Rouleau JL, Sweitzer NK (2018). Atrial fibrillation in heart failure with preserved ejection fraction: The TOPCAT trial. JACC Heart Fail.

[158] Lopes RD, Rordorf R, De Ferrari GM, Leonardi S, Thomas L, Wojdyla DM, Ridefelt P, Lawrence JH, De Caterina R, Vinereanu D (2018). Digoxin and mortality in patients with atrial fibrillation. J Am Coll Cardiol.

[159] Di Biase L, Mohanty P, Mohanty S, Santangeli P, Trivedi C, Lakkireddy D, Reddy M, Jais P, Themistoclakis S, Dello Russo A (2016). Ablation versus amiodarone for treatment of persistent atrial fibrillation in patients with congestive heart failure and an implanted device: results from the AATAC multicenter randomized trial. Circulation.

[160] Marrouche NF, Brachmann J, Andresen D, Siebels J, Boersma L, Jordaens L, Merkely B, Pokushalov E, Sanders P, Proff J (2018). Catheter ablation for atrial fibrillation with heart failure. N Engl J Med.

[161] Yang W-Y, Du X, Jiang C, He L, Fawzy AM, Wang L, Liu C, Xia S-J, Chang S-S, Guo X-Y (2020). The safety of discontinuation of oral anticoagulation therapy after apparently successful atrial fibrillation ablation: a report from the Chinese Atrial Fibrillation Registry study. Europace.

[162] Liang B, Zhao Y-X, Gu N. Discontinuation of oral anticoagulation therapy after apparently successful atrial fibrillation ablation. Europace. 2020. 10.1093/europace/euaa038 32091574

[163] Piccini JP, Connolly SJ, Abraham WT, Healey JS, Steinberg BA, Al-Khalidi HR, Dignacco P, van Veldhuisen DJ, Sauer WH, White M (2018). A genotype-directed comparative effectiveness trial of Bucindolol and metoprolol succinate for prevention of symptomatic atrial fibrillation/atrial flutter in patients with heart failure: rationale and design of the GENETIC-AF trial. Am Heart J.

[164] Praz F, Grasso C, Taramasso M, Baumbach A, Piazza N, Tamburino C, Windecker S, Maisano F, Prendergast B (2019). Mitral regurgitation in heart failure: time for a rethink. Eur Heart J.

[165] Obadia J-F, Messika-Zeitoun D, Leurent G, Iung B, Bonnet G, Piriou N, Lefèvre T, Piot C, Rouleau F, Carrié D (2018). Percutaneous repair or medical treatment for secondary mitral regurgitation. N Engl J Med.

[166] Iung B, Armoiry X, Vahanian A, Boutitie F, Mewton N, Trochu J-N, Lefèvre T, Messika-Zeitoun D, Guerin P, Cormier B (2019). Percutaneous repair or medical treatment for secondary mitral regurgitation: outcomes at 2 years. Eur J Heart Fail.

[167] Stone GW, Lindenfeld J, Abraham WT, Kar S, Lim DS, Mishell JM, Whisenant B, Grayburn PA, Rinaldi M, Kapadia SR (2018). Transcatheter mitral-valve repair in patients with heart failure. N Engl J Med.

[168] Pan W, Zhou D, Wu Y, Guo Y, Pan X, Pan C, Wei L, Ge J, Jilaihawi H, Leon MB (2019). First-in-human results of a novel user-friendly transcatheter edge-to-edge mitral valve repair device. JACC Cardiovasc Interv.

[169] Rosano GMC, Tamargo J, Kjeldsen KP, Lainscak M, Agewall S, Anker SD, Ceconi C, Coats AJS, Drexel H, Filippatos G (2018). Expert consensus document on the management of hyperkalaemia in patients with cardiovascular disease treated with renin angiotensin aldosterone system inhibitors: coordinated by the Working Group on Cardiovascular Pharmacotherapy of the European Society of Cardiology. Eur Heart J Cardiovasc Pharmacother.

[170] Pitt B, Anker SD, Bushinsky DA, Kitzman DW, Zannad F, Huang IZ (2011). Evaluation of the efficacy and safety of RLY5016, a polymeric potassium binder, in a double-blind, placebo-controlled study in patients with chronic heart failure (the PEARL-HF) trial. Eur Heart J.

[171] Pitt B, Bakris GL, Weir MR, Freeman MW, Lainscak M, Mayo MR, Garza D, Zawadzki R, Berman L, Bushinsky DA (2018). Long-term effects of patiromer for hyperkalaemia treatment in patients with mild heart failure and diabetic nephropathy on angiotensin-converting enzymes/angiotensin receptor blockers: results from AMETHYST-DN. ESC Heart Fail.

[172] Pitt B, Bushinsky DA, Kitzman DW, Ruschitzka F, Metra M, Filippatos G, Rossignol P, Du Mond C, Garza D, Berman L (2018). Evaluation of an individualized dose titration regimen of patiromer to prevent hyperkalaemia in patients with heart failure and chronic kidney disease. ESC Heart Fail.

[173] Ash SR, Singh B, Lavin PT, Stavros F, Rasmussen HS (2015). A phase 2 study on the treatment of hyperkalemia in patients with chronic kidney disease suggests that the selective potassium trap, ZS-9, is safe and efficient. Kidney Int.

[174] Fishbane S, Ford M, Fukagawa M, McCafferty K, Rastogi A, Spinowitz B, Staroselskiy K, Vishnevskiy K, Lisovskaja V, Al-Shurbaji A (2019). A phase 3b, randomized, double-blind, placebo-controlled study of sodium zirconium cyclosilicate for reducing the incidence of predialysis hyperkalemia. J Am Soc Nephrol.

[175] Spinowitz BS, Fishbane S, Pergola PE, Roger SD, Lerma EV, Butler J, von Haehling S, Adler SH, Zhao J, Singh B (2019). Sodium zirconium cyclosilicate among individuals with hyperkalemia: a 12-month phase 3 study. Clin J Am Soc Nephrol.

[176] Ding N, Zhang X (2018). Transvenous phrenic nerve stimulation, a novel therapeutic approach for central sleep apnea. J Thorac Dis.

[177] Cowie MR, Woehrle H, Wegscheider K, Angermann C, d’Ortho M-P, Erdmann E, Levy P, Simonds AK, Somers VK, Zannad F (2015). Adaptive servo-ventilation for central sleep apnea in systolic heart failure. N Engl J Med.

[178] Ponikowski P, Javaheri S, Michalkiewicz D, Bart BA, Czarnecka D, Jastrzebski M, Kusiak A, Augostini R, Jagielski D, Witkowski T (2012). Transvenous phrenic nerve stimulation for the treatment of central sleep apnoea in heart failure. Eur Heart J.

[179] Abraham WT, Jagielski D, Oldenburg O, Augostini R, Krueger S, Kolodziej A, Gutleben K-J, Khayat R, Merliss A, Harsch MR (2015). Phrenic nerve stimulation for the treatment of central sleep apnea. JACC Heart Fail.

[180] Zhang X-L, Ding N, Ni B, Yang B, Wang H, Zhang S-J (2017). Safety and feasibility of chronic transvenous phrenic nerve stimulation for treatment of central sleep apnea in heart failure patients. Clin Respir J.

[181] Collaborators GDaIIaP (2018). Global, regional, and national incidence, prevalence, and years lived with disability for 354 diseases and injuries for 195 countries and territories, 1990–2017: a systematic analysis for the Global Burden of Disease Study 2017. Lancet..

[182] Taylor CJ, Ordóñez-Mena JM, Roalfe AK, Lay-Flurrie S, Jones NR, Marshall T, Hobbs FDR (2019). Trends in survival after a diagnosis of heart failure in the United Kingdom 2000–2017: population based cohort study. BMJ.

[183] Benjamin EJ, Virani SS, Callaway CW, Chamberlain AM, Chang AR, Cheng S, Chiuve SE, Cushman M, Delling FN, Deo R et al. Heart disease and stroke statistics—2018 update: a report from the American Heart Association. Circulation. 2018;137(12). 10.1161/CIR.0000000000000558 29386200

[184] Benjamin EJ, Muntner P, Alonso A, Bittencourt MS, Callaway CW, Carson AP, Chamberlain AM, Chang AR, Cheng S, Das SR et al. Heart disease and stroke statistics—2019 update: a report from the American Heart Association. Circulation. 2019;139(10). 10.1161/CIR.0000000000000659 30700139

[185] Cook C, Cole G, Asaria P, Jabbour R, Francis DP (2014). The annual global economic burden of heart failure. Int J Cardiol.

[186] Schiattarella GG, Tong D, Hill JA (2020). Can HFpEF and HFrEF coexist?. Circulation.

[187] Redfield MM (2016). Heart failure with preserved ejection fraction. N Engl J Med.

[188] Sharma K, Kass DA (2014). Heart failure with preserved ejection fraction: mechanisms, clinical features, and therapies. Circ Res.

[189] Gheorghiade M, Greene SJ, Butler J, Filippatos G, Lam CSP, Maggioni AP, Ponikowski P, Shah SJ, Solomon SD, Kraigher-Krainer E (2015). Effect of vericiguat, a soluble guanylate cyclase stimulator, on natriuretic peptide levels in patients with worsening chronic heart failure and reduced ejection fraction: the SOCRATES-REDUCED randomized trial. JAMA.

[190] Pieske B, Maggioni AP, Lam CSP, Pieske-Kraigher E, Filippatos G, Butler J, Ponikowski P, Shah SJ, Solomon SD, Scalise A-V (2017). Vericiguat in patients with worsening chronic heart failure and preserved ejection fraction: results of the SOluble guanylate Cyclase stimulatoR in heArT failurE patientS with PRESERVED EF (SOCRATES-PRESERVED) study. Eur Heart J.

[191] Armstrong PW, Roessig L, Patel MJ, Anstrom KJ, Butler J, Voors AA, Lam CSP, Ponikowski P, Temple T, Pieske B (2018). A multicenter, randomized, double-blind, placebo-controlled trial of the efficacy and safety of the oral soluble guanylate cyclase stimulator: The VICTORIA trial. JACC Heart Fail..

[192] Pieske B, Patel MJ, Westerhout CM, Anstrom KJ, Butler J, Ezekowitz J, Hernandez AF, Koglin J, Lam CSP, Ponikowski P (2019). Baseline features of the VICTORIA (Vericiguat global study in subjects with heart failure with reduced ejection Fraction) trial. Eur J Heart Fail.

[193] Armstrong PW, Pieske B, Anstrom KJ, Ezekowitz J, Hernandez AF, Butler J, Lam CSP, Ponikowski P, Voors AA, Jia G et al. Vericiguat in patients with heart failure and reduced ejection fraction. N Engl J Med. 2020.

[194] Hasenfuß G, Hayward C, Burkhoff D, Silvestry FE, McKenzie S, Gustafsson F, Malek F, Van der Heyden J, Lang I, Petrie MC (2016). A transcatheter intracardiac shunt device for heart failure with preserved ejection fraction (REDUCE LAP-HF): a multicentre, open-label, single-arm, phase 1 trial. Lancet.

[195] Cattadori G, Segurini C, Picozzi A, Padeletti L, Anzà C (2018). Exercise and heart failure: an update. ESC Heart Fail.

[196] Kitzman DW, Brubaker P, Morgan T, Haykowsky M, Hundley G, Kraus WE, Eggebeen J, Nicklas BJ (2016). Effect of caloric restriction or aerobic exercise training on peak oxygen consumption and quality of life in obese older patients with heart failure with preserved ejection fraction: a randomized clinical trial. JAMA.

[197] Antonicelli R, Spazzafumo L, Scalvini S, Olivieri F, Matassini MV, Parati G, Del Sindaco D, Gallo R, Lattanzio F (2016). Exercise: a “new drug” for elderly patients with chronic heart failure. Aging.

[198] Panagopoulou N, Karatzanos E, Dimopoulos S, Tasoulis A, Tachliabouris I, Vakrou S, Sideris A, Gratziou C, Nanas S (2017). Exercise training improves characteristics of exercise oscillatory ventilation in chronic heart failure. Eur J Prev Cardiol.

[199] Snoek JA, Eijsvogels TMH, Van ‘t Hof AWJ, Prescott E, Hopman MT, Kolkman E, De Kluiver EDP (2018). Impact of a graded exercise program on VO_2_ peak and survival in heart failure patients. Med Sci Sports Exerc.

[200] Taylor R, Walker S, Ciani O, Warren F, Smart N, Piepoli M, Davos C (2019). Exercise-based cardiac rehabilitation for chronic heart failure: the EXTRAMATCH II individual participant data meta-analysis. Health Technol Assess.

[201] Hegde SM, Claggett B, Shah AM, Lewis EF, Anand I, Shah SJ, Sweitzer NK, Fang JC, Pitt B, Pfeffer MA (2017). Physical activity and prognosis in the TOPCAT trial (Treatment of Preserved Cardiac Function Heart Failure With an Aldosterone Antagonist). Circulation.

